# Recent progress in chemosensors based on pyrazole derivatives

**DOI:** 10.1039/d0ra02394a

**Published:** 2020-05-22

**Authors:** Alexis Tigreros, Jaime Portilla

**Affiliations:** Department of Chemistry, Bioorganic Compounds Research Group, Universidad de los Andes Carrera 1 No. 18A-10 Bogotá 111711 Colombia jportill@uniandes.edu.co

## Abstract

Colorimetric and fluorescent probes based on small organic molecules have become important tools in modern biology because they provide dynamic information concerning the localization and quantity of the molecules and ions of interest without the need for genetic engineering of the sample. In the past five years, these probes for ions and molecules have attracted great attention because of their biological, environmental and industrial significance combined with the simplicity and high sensitivity of absorption and fluorescence techniques. Moreover, pyrazole derivatives display a number of remarkable photophysical properties and wide synthetic versatility superior to those of other broadly used scaffolds. This review provides an overview of the recent (2016–2020) findings on chemosensors containing pyrazole derivatives (pyrazoles, pyrazolines and fused pyrazoles). The discussion focuses on the design and physicochemical properties of chemosensors in order to realize their full potential for practical applications in environmental and biological monitoring (sensing of metal ions, anions, explosives, and biomolecules). We also present our conclusions and outlook for the future.

## Abstract 2

This review provides an overview on the recent (2016–2020) findings on chemosensors based on pyrazole derivatives. The discussion focuses on the design, physicochemical properties and practical applications of probes.

## Graphical abstract

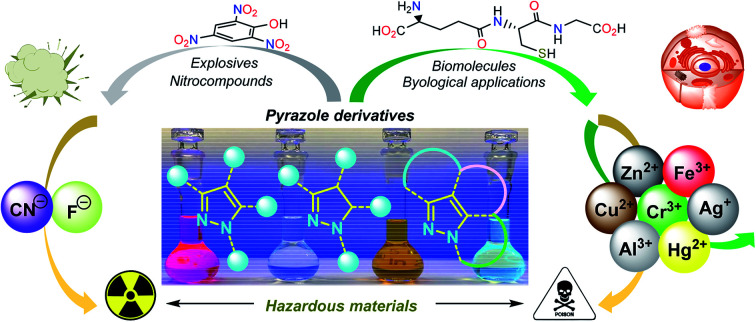

## 1. Introduction

**Fig. 1:**
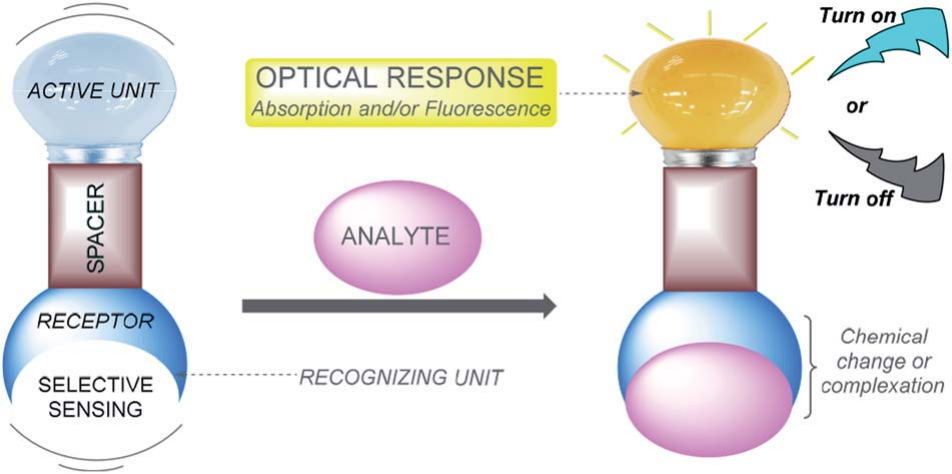
Schematic representation of a colorimetric/fluorescent chemosensor.

The sensing of ions *via* molecular recognition is one of the most active and exciting research fields in analytical chemistry, due to its vital role in industrial processes, environmental science, medicine, catalysis, and biological and human sciences.^1,2^ Chemosensors are molecules able to selectively and reversibly bind (if not, chemodosimeter is the proper name) the analyte of interest with a concomitant change in one or more properties of the system, such as redox potential or absorption or fluorescence spectra.^3^ Because of the two different processes occurring during analyte detection, that is, molecular recognition and signal transduction, chemosensors can usually be schematized as made of three possible different components (Fig. 1): a receptor (responsible for the selective analyte binding), an active unit (whose properties should change upon binding) and, eventually, a spacer that can change the geometry of the system and tune the electronic interaction between the two aforementioned moieties.^4^

Based on different photophysical processes, conventional sensing mechanisms, including photoinduced electron transfer (PET),^5^ intramolecular charge transfer (ICT),^6^ metal–ligand charge transfer (MLCT),^7^ twisted intramolecular charge transfer (TICT),^8^ electronic energy transfer (EET),^9^ and fluorescence resonance energy transfer (FRET),^10^ have been used for analyte detection. Whenever a molecular probe is being designed for a particular analyte, three crucial factors need to be considered: (1) high sensitivity (detection limit), (2) high specificity (distinguishing among metal ions), and (3) high selectivity (for the specific ion pool under investigation). In addition, it might not be necessary to monitor metal ions at low concentrations but rather over a dynamic range of concentrations.^11^ Designing an effective chemosensor for the detection of an analyte with both selectivity and sensitivity properties is a challenging research problem and is expected to offer major biological and environmental benefits.

**Fig. 2:**
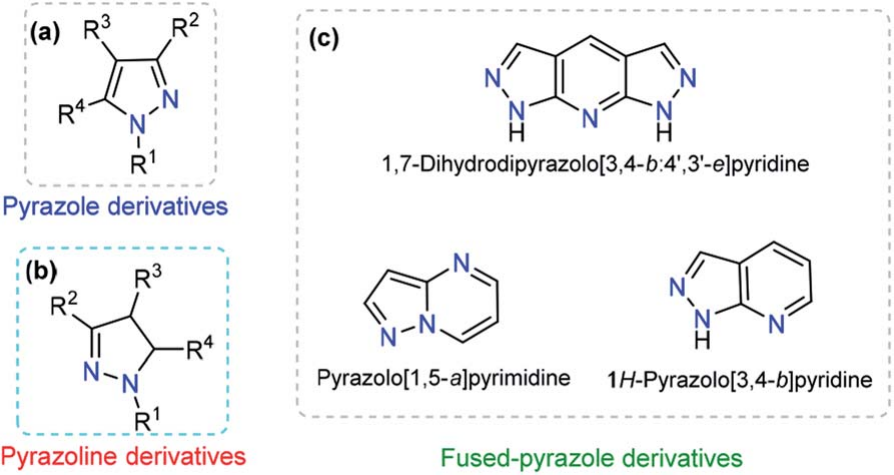
General structure of (a) pyrazole, (b) pyrazoline and (c) some fused-pyrazoles.

Pyrazole is a 5-membered heteroaromatic compound containing two adjacent nitrogen atoms (Fig. 2a). NH-pyrazoles can act as both weak bases and moderately weak acids because they have a pyridine-type proton-acceptor (*i.e.*, cation-receptor) nitrogen atom (C=N) and one pyrrole-type nitrogen atom (N–H) with proton-donor behavior. The versatility of pyrazole derivatives in synthetic^12^ and/or biological^13^ applications has been well documented,^14^ being even one of the most studied compounds among the azole family,^15^ although examples of natural products containing the pyrazole moiety are very scarce.^16^ Pyrazole by itself does not exhibit any fluorescent properties. However, the photophysical properties of appropriately substituted pyrazoles have been studied, and remarkable results have been found, including high fluorescence quantum yields, notable solvatochromic behavior, high Stokes shifts, nonlinear optical properties, and so on.^17–19^

The dihydro-derivative of pyrazole (pyrazoline) is another important aza-heterocycle that has been extensively researched (Fig. 2b). The ring is quite stable and has inspired chemists to carry out various structural variations in the ring. This has propelled the development of diverse pyrazolines with an array of pharmacological activities, *viz.*, anti-inflammatory,^20^ analgesic,^21^ antimicrobial,^22^ anticancer,^23^ and antidepressant activities,^24^ among others. In addition to these biological applications, a number of pyrazolines have been extensively used in materials sciences as solvatochromic probes,^25–27^ electroluminescent compounds,^28–30^ chemosensors, and nonlinear optical (NLO) materials.^31,32^

Dye photophysical properties are closely related to the planar structures, π-extended conjugation and nature of heteroatoms. Consequently, fused pyrazoles represent an attractive scaffold in organic optoelectronic materials. Developing new methodologies related to the synthesis of fused pyrazoles has been a regular topic in organic chemistry,^12^ and derivatives with special photophysical properties have been synthesized in recent years, including indazoles,^33^ naphtho[2,1-*d*]-1*H*-pyrazoles,^34^ pyrazolo–pyrrolo-pyrimidines,^35^ polycyclic fused pyrazoles,^36^ pyrazolo[3,4-*b*]pyridine-based coumarins,^37^ pyrazole[3,4-*b*]thieno[2,3-*e*]pyridines,^38^ and bis-pyrazolo[3,4-*b*:4′,3′-*e*]pyridines.^39^ The number and type of heteroatoms play a paramount role in the selectivity of the probe. Moreover, the photophysical properties and chemo-sensing abilities are closely related to the heteroatom position (Fig. 2c).^37^

The goal of this review is to highlight some of the more recent developments of colorimetric and fluorescent pyrazole-based probes that have been employed for sensing cations (Ag^+^, Cu^2+^, Zn^2+^, Hg^2+^, Al^3+^, Fe^3+^, and Cr^3+^), anions (F^−^ and CN^−^) or molecules (picric acid, glutathione and tryptamine), in which a chemical reaction or physical interaction produces a significant change in the photophysical properties. In addition, the biological applications of these probes based on pyrazole derivatives are discussed by means of meeting the requirements of real applications.

## 2. Probes for ions based on pyrazole derivatives

### 2.1. Cations

The detection of heavy and transition metals has attracted much attention, and traditional detection methods for metal ions such as inductively coupled plasma atomic emission spectrometry (ICP-AES),^40–42^ atomic absorption spectroscopy (AAS),^43,44^ high-performance liquid chromatography,^45^ fluorescence techniques^46^ and electrochemical methods (EMs)^47,48^ require sophisticated equipment and tedious sample preparation procedures. In addition, these analytical instruments are expensive and require trained operators.^49^ Therefore, it is not surprising that the development of an alternative method for rapid and facile detection of metals has attracted tremendous attention from chemists, biologists and environmentalists. In meeting these challenges, colorimetric and fluorescence-based chemosensors may play a vital role that allows a quick response time, high sensitivity and good selectivity.^50^

A number of organic molecules containing a wide range of donor sites have been reported for heavy metal detection by colorimetric and fluorescent techniques.^4^ Among these molecules, pyrazole and its derivatives are an important class of chelating ligands for metal atoms. The combination of pyrazole derivative with different functional moieties strengthens the complexing behavior and/or photophysical properties and allows the moieties to act as good candidates for complexation with a wide range of metals due to its higher number of nitrogen, sulfur and oxygen donor sites. In this section, we present a recent advance in metal detection through colorimetric and fluorescence pyrazole derivatives based on the chemosensor approach.

#### 2.1.1. Copper(ii)

Copper (Cu) is the third most abundant transition metal in the human body and plays pivotal roles as a trace element in many biological systems processes. However, the presence of this metal in excess concentrations in living organisms may cause fatal health issues. Thus, the detection of Cu^2+^ is a relevant aim in the colorimetric/fluorescence research field.^51,52^ Normally, copper is present in nature as a divalent cation (Cu^2+^) and can react with a number of ligands in the formation of a variety of complexes with tunable photophysical properties.

**Fig. 3:**
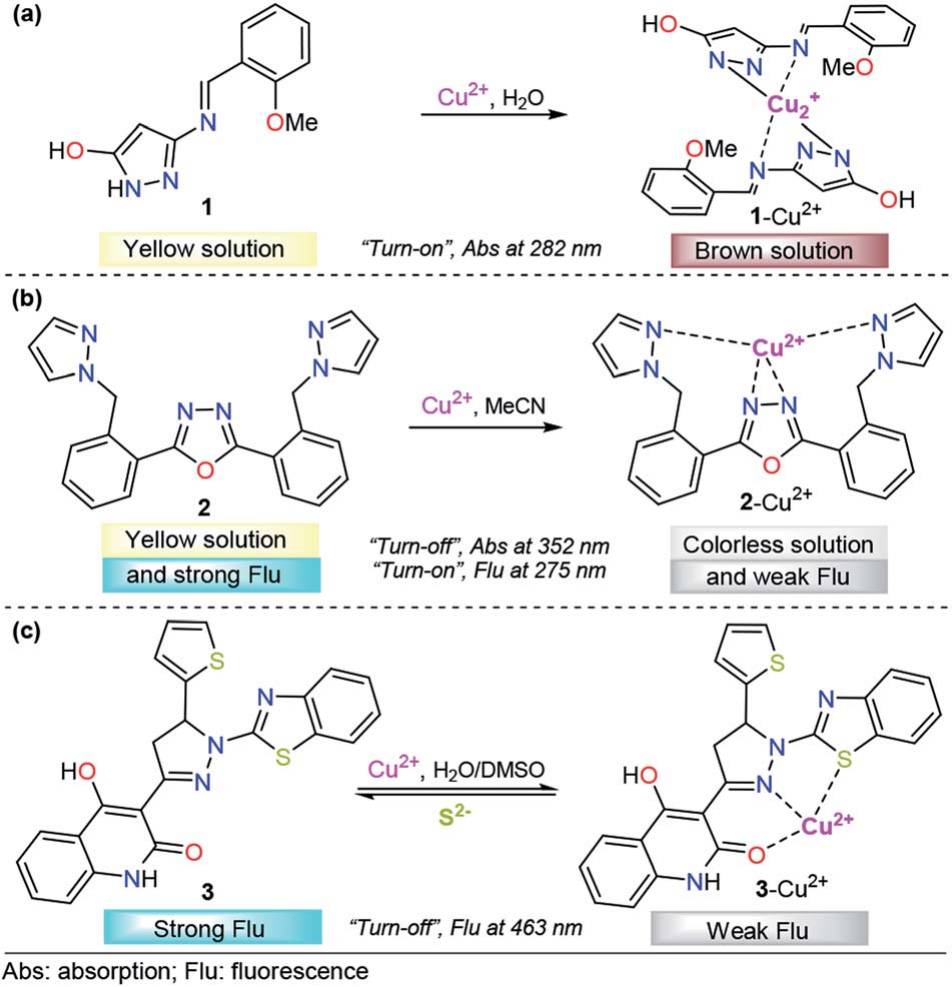
Pyrazole-based probe for Cu^2+^ chemosensing. Three derivatives of (a) NH-pyrazole, (b) bis-pyrazole 2 and (c) pyrazoline 3 are shown.

Encouraged by the need for low-cost and simple molecules for copper sensing, Armaković *et al.* developed a small-molecule colorimetric chemodosimeter for Cu^2+^ detection by using azomethine-pyrazole derivative 1 (Fig. 3a).^53^ Upon the addition of Cu^2+^ into solutions of 1, the intensity of the absorption band at 282 nm increased as a result of complex formation between compound 1 and Cu^2+^. A low limit of detection (LOD) and selectivity toward other common cations were observed (1.6 μM). A 2 : 1 ligand/metal relationship was proposed and evidenced by mass spectrometry. Moreover, density functional theory (DFT) calculations of the bond dissociation energies for hydrogen abstraction (H-BDE) indicate that 1 ligand is highly sensitive toward autoxidation. The pyrazole moiety serves as a coordination point by binding the metal ion through one of its nitrogen atoms.

Among the diverse chemosensors, fluorescent probes present many advantages: fluorescence measurements are usually very sensitive (even single molecule detection is possible, but only under special conditions), inexpensive, easily performed and versatile, offering subnanometer spatial resolution with submillisecond temporal resolution and submicron visualization. The versatility of fluorescent probes also originates from the wide number of parameters that can be tuned in order to optimize the convenient signal.^54^ Regarding the properties, the paramagnetic behavior of Cu^2+^ has been broadly exploited in the chemosensors area, due to its well-known quenching of fluorescent species. This property has inspired the design of various fluorescent probes for this cation.^55–57^

Accordingly, pyrazole has been combined with some fluorescent groups with electron-rich atoms to bind and consequently detect Cu^2+^ ions through a “*turn-off*” pathway. A successful example was presented by Wang and his coworkers combining a flexible pyrazole with an oxazole derivative,^58^ a well-known fluorescent group (Fig. 3b).^59,60^ The addition to Cu^2+^ enhanced the absorbance intensity of 2, at approximately 275 nm, by 2-fold in acetonitrile. On the other hand, chemodosimeter 2 emitted blue fluorescence (*λ*_em_ = 352 nm), which was only quenched by increasing the Cu^2+^ ion concentration. The fluorescence intensity of the sensor showed a linear response to Cu^2+^ in the concentration range of 0–15 μM, with a LOD of 2.14 μM. Moreover, a fast response (10 s) of this compound was observed, meeting the requirement of real-time detection. Fluorescence titration, ^1^H NMR experiments, ESI-MS analysis and theoretical calculations confirmed that the fluorescence quenching is caused by complex formation between ligand 2 and Cu^2+^ with 1 : 1 stoichiometry, in which the quenching of the fluorescence could be attributed to the paramagnetic properties of Cu^2+^. Despite the poor fluorescent properties of a single pyrazole, its binding ability was highly important in the formation of complex 2–Cu^2+^.

Following the same design guide, Mohan *et al.* used a quinoline–pyrazoline–benzothiazole module, compound 3 (Fig. 3c).^61^ Optical studies were carried out in H_2_O–DMSO (9 : 1 v/v; pH 7.4 in PBS buffer). In the absence of Cu^2+^ ions, probe 3 exhibits a broad band at 377 nm. Coordination of copper ions to 3 resulted in the formation of a new absorption band at 386 nm and synchronously small decrease in the absorption band intensity. However, this change was very small for analytical applications. On the other hand, probe 3 showed strong fluorescence emission at 463 nm (*ϕ* = 0.4398), when excited at 370 nm. After the addition of 1 equivalent of Cu^2+^, a significant decrease in the fluorescence intensity was observed (*ϕ* = 0.020), and from fluorescence titration experiments of 3 with Cu^2+^, the LOD was calculated to be 0.16 nM. Furthermore, as is well known, Cu^2+^ can coordinate with S^2−^ anions to form the highly stable species CuS. Taking advantage of this fact, the authors proposed and studied the complex 3–Cu^2+^ as a sulfide chemosensor and found excellent performance: high selectivity toward other common anions and a LOD as low as 0.2 μM. Fluorescence imaging experiments with MG-63 cell lines exclusively demonstrate the applicability of probe 3 in Cu^2+^ imaging in biological systems. The probe was also used for the real-time analysis of S^2−^ ions in tannery effluent for environmental monitoring. From the DFT results, plausible coordination of 3 with Cu^2+^ was found, and an important role of benzothiazolyl, pyrazolyl and quinoline groups in the formation of this complex was noticed.

**Fig. 4:**
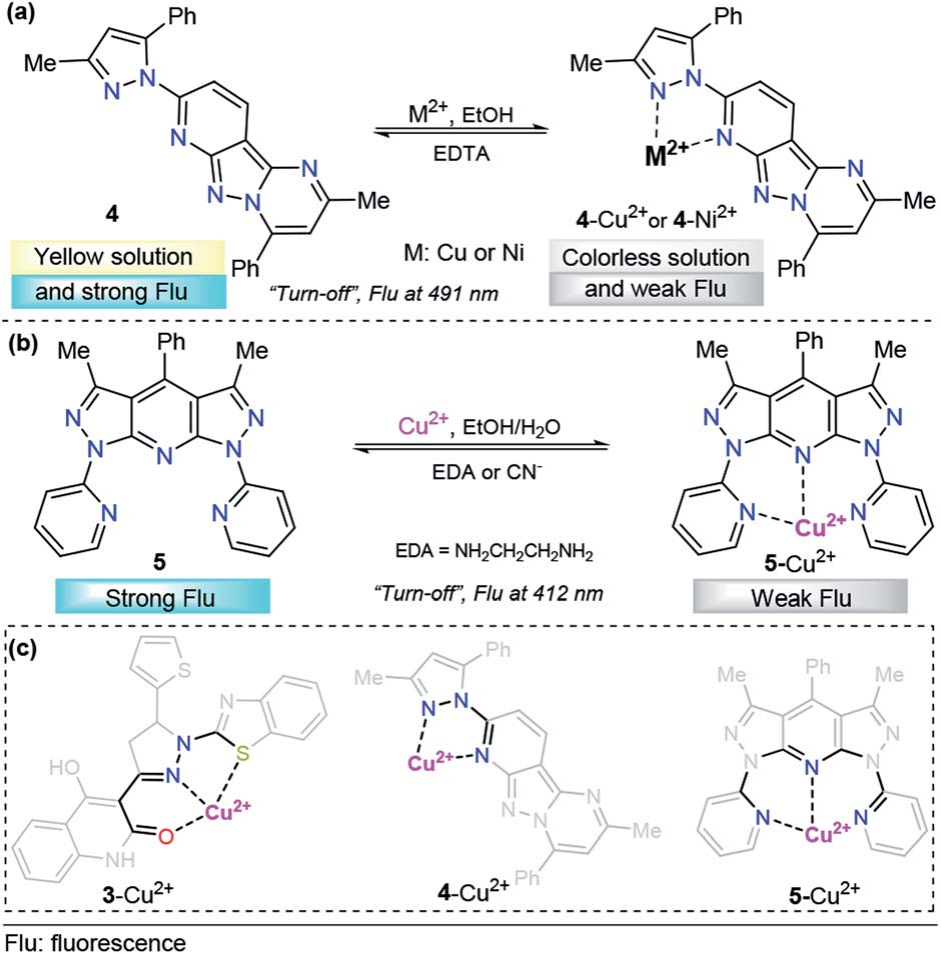
Probes for Cu^2+^ sensing based on fused pyrazoles (a) 4 and (b) 5. (c) Structural relationship between the pyrazole-based probe for Cu^2+^ sensing.

To the best of our knowledge, in the last five years, polycyclic aromatics containing pyrazole cores have rarely been investigated for copper(ii) sensing. A representative example was the tridentate pyrazolopyrimidine-based chemosensor 4 for the detection of Cu^2+^ and Ni^2+^, developed by Hu's research group (Fig. 4a).^62^ The emission spectroscopy of 4 and its fluorescence titration with a number of metal salts were performed in ethanol. Probe 4 displays an emission band at 491 nm with strong fluorescence (*ϕ* = 0.228) with an excitation wavelength (*λ*_exc_) of 300 nm. A solution of 4 was titrated with aqueous M^2+^ (M = Cu or Ni), and the fluorescence emission intensity at 491 nm was gradually quenched (down to *ϕ* = 0.036 for 4–Cu^2+^ and *ϕ* = 0.023 for 4–Ni^2+^). These titrations with Cu^2+^ or Ni^2+^ ions displayed good linearity in the concentration range of 0–10.8 μM and 0–8.4 μM, respectively. The LOD for each cation was calculated to be 0.043 μM for Cu^2+^ and 0.038 μM for Ni^2+^. In the presence of Na_2_EDTA, reversibility of the complexes 4–Cu^2+^and 4–Ni^2+^ was found, allowing the reuse of the probe. Further studies allowed the authors to conclude a 1 : 1 stoichiometric relationship of 4 with the metals, and single-crystal analysis revealed that both complexes are mononuclear with a ligand/metal ratio of 1 : 1. The crystallographic studies also shown nonparticipation of the nitrogen atom of the fused pyrazole in the coordination complex, so this compound acts as a bidentate ligand. DFT calculations suggested that the fluorescence quenching mechanism in 4–Cu^2+^ and 4–Ni^2+^ is caused by an ICT phenomenon. Finally, laser scanning confocal microscopy experiments at 300 nm showed that 4 can be employed for the detection of Cu^2+^ and Ni^2+^ in living cells such as T-24 cells.

As part of our continuing research in the chemosensor field,^63^ we recently designed and synthesized a novel 1,7-dipyridyl-bis-pyrazolo[3,4-*b*:4′,3′-*e*]pyridine probe for Cu^2+^ sensing, ligand 5 (Fig. 4b).^64^ The UV-vis spectrum was characterized by an absorption band at 250 nm when using 99 : 1 ethanol/water as the solvent; however, this compound showed no significant absorption changes with any of the cations tested. Probe 5 exhibited an intense emission band at 412 nm (*ϕ* = 0.500, *λ*_exc_ = 250 nm). Upon the addition of different cations, probe 5 displayed good selectivity toward biologically and environmentally important ions, such as Cu^2+^, Co^2+^, Ni^2+^ and Hg^2+^. Among these cations, ligand 5 showed a notable selectivity for Cu^2+^. Then, the authors studied the chemo-sensing properties of 5 by adding 0.2 to 100 equivalents of each metal to 1.00 μM solutions of fluorescent compound 5. In accordance with the results, low LODs were obtained for these metals (0.026, 0.124, 0.157 and 0.837 μM for Cu^2+^, Co^2+^, Ni^2+^ and Hg^2+^, respectively). Notably, probe 5 was found to reversibly bind Cu^2+^ ions, as tested by reacting with ethylenediamine (EDA). By using high-resolution mass spectrometry (HRMS) analysis, the formation of 5–Cu^2+^ complex was evidenced. In addition to the well-known paramagnetic properties of Cu^2+^ that deactivate excited states, we proposed a plausible mechanism for fluorescence quenching in which a TICT process is key for this type of ligand. Finally, preliminary studies indicated that complex 5–Cu^2+^ is able to detect cyanide (CN^−^) ions with a LOD as low as 0.324 μM. These pyrazole derivatives could be useful moieties in designing systems for water treatment based on resins properly functionalized with 5-type compounds, and research is being conducted in this regard.

It is important to note that the 5- or 6-membered ring is a common factor in the formation of probe–Cu^2+^ complexes, with the exception of probe 2, which has a flexible binding site. In all cases, the azole displays a versatile role in the binding site by contributing coordination points for the metal and/or allowing the correct geometry of the probe, as highlighted in Fig. 4c.

#### 2.1.2. Zinc(ii)

Zinc (Zn) is the second most abundant d-block metal in the human body and is often found as pools of mobile ions in specific tissues of the body.^65^ In the past decade, fluorescent chemosensors for Zn^2+^ ions have attracted great attention because of the biological significance of zinc combined with the simplicity and high sensitivity of fluorescence assays.^66–68^ Transitions ions with closed-shell d-orbitals, such as Zn^2+^, do not introduce low-energy metal-centered or charge-separated excited states into the molecule, so energy and electron transfer processes cannot take place. Thus, when Zn^2+^ is added, the fluorescence intensity of their complexes tends to increase,^69^ so the interaction of Zn^2+^ ions with pyrazolines follows a “*turn-on*” behavior in terms of the fluorescence properties. The mechanism proposed for pyrazolines considers the role of many processes: (1) PET inhibition, (2) chelation-enhanced fluorescence (CHEF), (3) restriction of the hydrazone moiety isomerization, and (4) excited-state intramolecular proton transfer (ESIPT).

**Fig. 5:**
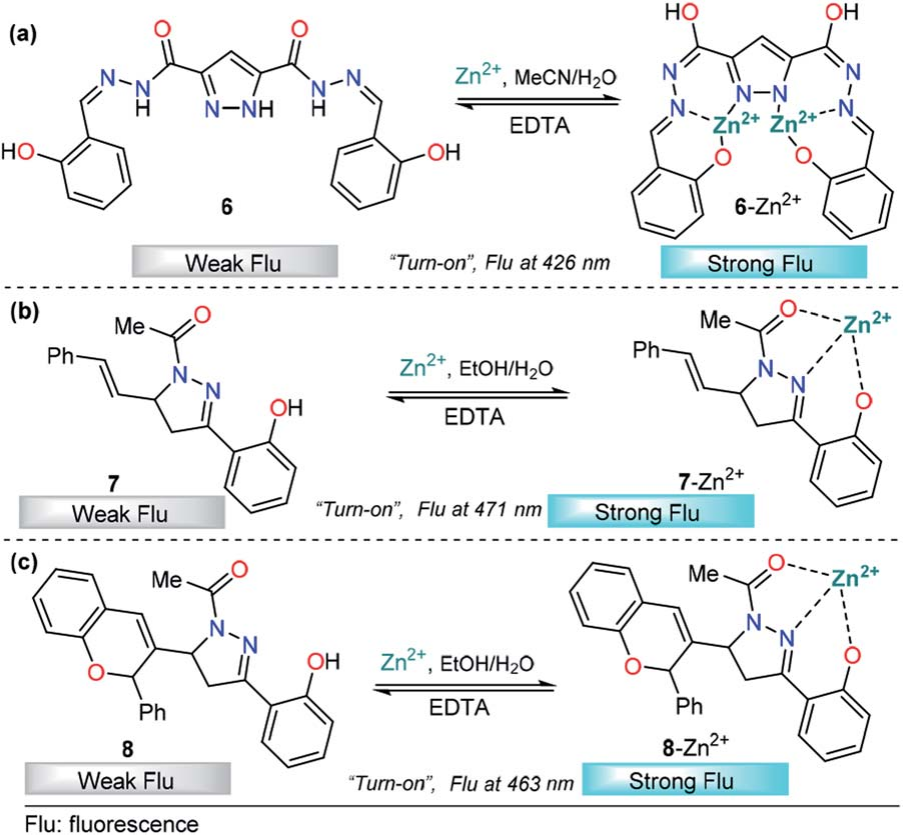
(a) Pyrazoles and (b and c) pyrazolines in Zn^2+^ sensing.

Recently, azoles such as substituted pyrazole have been broadly used for these purposes, achieving very good results.^70^ A representative example is the study by V. Revankar and K. Naik in which they synthesized a pyrazole equipped with carbohydrazide and hydroxybenzylidene groups, compound 6 (Fig. 5a).^71^ The absorption bands in DMF/water (1 : 1, v/v pH = 7.4) displayed by 6 are clearly affected only in the presence of Zn^2+^ ions. The solution turned from colorless to yellow due to a new absorption band at 385 nm. The free form of 6 shows moderate fluorescence (*ϕ* = 0.026) with a peak at 426 nm (*λ*_exc_ = 330 nm), and increases linearly up to twofold in the presence of Zn^2+^. The LOD of 6 for Zn^2+^ was found to be as low as 992 nM. Further, low interference was noticed in the presence of other cations with similar properties such as Cd^2+^. Moreover, the reversibility of the 6–Zn^2+^ complex with EDTA was achieved, showing that probe 6 can be reused a number of times. The efficacy of 6 to permeate cell membranes and detect intracellular zinc was evaluated by using HeLa cell cultures with excellent results; thus, these studies demonstrate the potential of probe 6 to detect selective Zn^2+^ ions at low concentrations, even in biological samples. By using ^1^H NMR titration experiments and theoretical calculations, the authors attributed the photophysical changes in compound 6 to an PET phenomenon inhibition and activation of an ICT process after Al-metal coordination.

In the past few years, pyrazolines have undoubtedly been one of the most studied chemosensors for Zn^2+^ sensing. Successful strategies have combined pyrazolines with aromatic and/or heteroaromatic groups at positions 1, 3 and/or 5 of the pyrazoline core. In particular, the use of groups with chelating properties, to build a proper binding site, has produced outstanding results. In 2014, Ting-Ting Zhang and coworkers showed the paramount importance of the 2-hydroxyl group in the substituent present at position 3 of the pyrazoline ring.^67^ On the other hand, Lorenzo Caggiano's group showed that replacing the aryloxy group with 2-pyridines and the acetyl group with a methyl group led to a low selectivity for zinc in the presence of Cd^2+^.^72^ Meanwhile, the acetyl group was successfully replaced with a carbothioamide^73^ or pyridazine^74^ group with excellent results. With these previous findings and motivated by interesting applications, Zhang explored this area of chemosensor development and published two closely related studies in 2018. In those studies, cinnamyl-pyrazoline 7 (Fig. 5b)^75^ and chromene–pyrazoline 8 (Fig. 5b)^76^ were employed for the selective determination of Zn^2+^. Small differences were noticed in the photophysical properties, *e.g.*, emission bands at 471 nm for 7 and 463 nm for 8 (*λ*_exc_ = 381 nm). In both cases, a mixture of ethanol/water was used as the solvent, and high selectivity toward other common ions was observed. In the presence of EDTA, the formation of complexes 7–Zn^2+^ and 8–Zn^2+^ was found to be reversible. In addition, the viability of probes 7 and 8 with Zn^2+^ has practical application in live cell imaging; however, the LOD for 8 (16.03 nM) was two orders of magnitude lower than that of 7 (0.29 nM). Since the LODs are closely related to fluorescence intensity, this result indicates that the introduction of groups with high intrinsic fluorescence may improve the LOD of the chemosensor. Studies developed by P.-S. Mohan and his research team corroborate the aforementioned assumption.

**Fig. 6:**
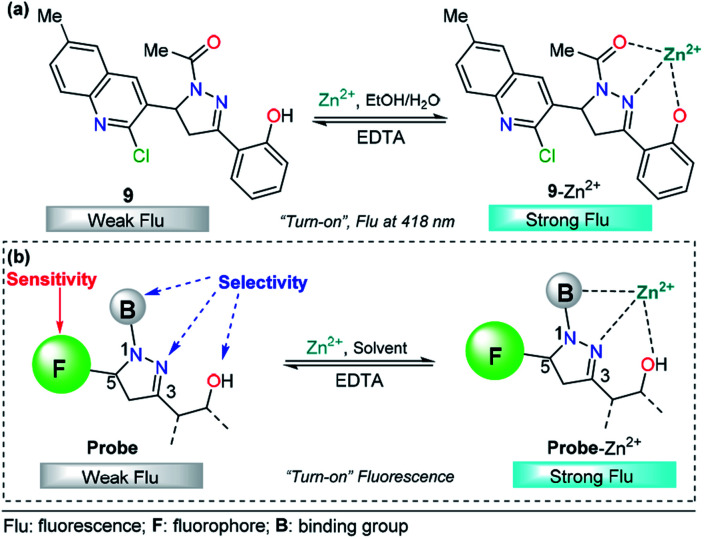
(a) Probe for Zn^2+^ sensing based on pyrazoline 4 and (b) structural requirements for improved Zn^2+^ ion sensing.

The pyrazoline described by Mohan's group is very similar to compounds 7 and 8. In this case, a quinoline group was placed at position 3 instead of a cinnamyl or chromene moiety, yielding compound 9.^77^ After conventional experiments, the authors showed that 9 can selectively detect Zn^2+^ in the presence of other related metal ions, and the viability of 9 was demonstrated by live-cell imaging. The LOD of this probe was found to be 2.9 nM. Thus, the high intensity observed in the emission spectrum of complex 9–Zn^2+^ plays an important role in this property, demonstrating that the nature of the group at position 3 is very important for the sensitivity of the probe (Fig. 6a).

Based on the above information, it is clear that a probe containing a pyrazoline center can be designed for selective Zn^2+^ ion detection. First, at position 1 of the ring, there must be an acetyl group or a related moiety; second, at position 5, a highly fluorescent group must be placed in order to improve the sensitivity; and third, a 2-aryloxy substituent will improve the selectivity toward Zn^2+^ ions (Fig. 6b).

#### 2.1.3. Mercury(ii)

Mercury (Hg) is one of the most prevalent toxic elements on earth, and arises from many sources such as gold production, coal plants, thermometers, barometers and mercury lamps.^78^ For the purposes of detection and quantitative determination of mercury ions, much effort has been devoted to the development of appropriate methods. In this sense, in recent years, a large number of colorimetric and fluorescent chemosensors have been developed for Hg^2+^ detection.^79–82^ The low cost, rapidity, deftness and applicability to the natural environmental milieu of the chemosensor approach support the large effort dedicated to this research field.^83–87^ Of course, tuning the pyrazole derivative properties has been a recurrent tool in the sensor design and successful detection and quantification of mercury(ii). In contrast to Cu^2+^, Zn^2+^ and Al^3+^ ions, which have a regular tendency in how they affect the fluorescence while interacting with different probes, mercury ions can participate in a variety of processes in which the fluorescence behavior can “*turn-off*” or “*turn-on*”, depending on the ligand used.

**Fig. 7:**
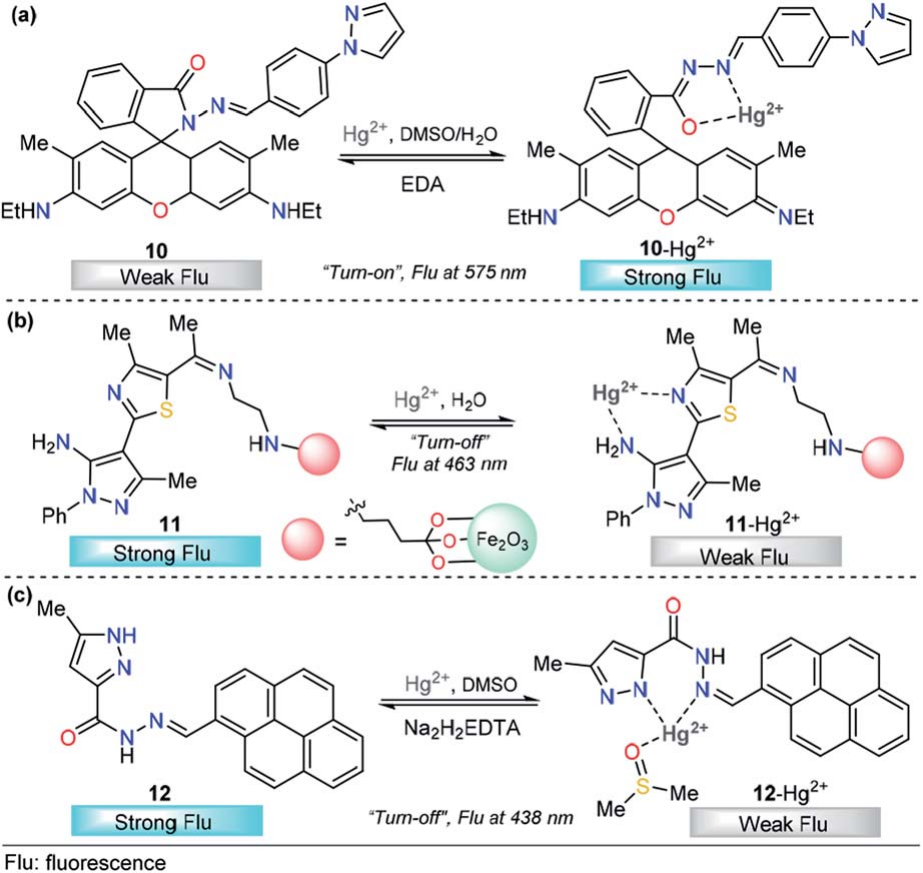
Pyrazole-based probe for Hg^2+^ chemosensing. Three derivatives of (a) *N*-arylpyrazole 10, (b) *N*-phenylpyrazole 11 and (c) NH-pyrazole 12 are shown.

The integration of rhodamine with pyrazole was explored by Alam *et al.* for metal sensing. In this example, the pyrazole plays an important role as a coordination point. However, low selectivity was achieved and Fe^3+^, Cr^3+^ and Al^3+^ can be incorporated in the binding site,^88^ a disadvantage for the quantification of a particular cation. Later, Wang and his research team successfully developed a new dual pyrazole-rhodamine 6G probe 10 that selectively detects Hg^2+^ ions in aqueous DMSO solution (Fig. 7a).^89^ Free sensor 10 exhibited one fluorescence emission band at about 575 nm (*λ*_exc_ = 525 nm), and after the addition of Hg^2+^ (1.0 equiv.), a significant fluorescence enhancement was observed, with the emission maximum at approximately 575 nm. Compound 10 showed high sensitivity toward Hg^2+^, with a LOD as low as 20.7 nM. Probe 10 has potential application value to detect Hg^2+^ within a biological scale of pH values, and excellent reversibility properties were revealed. The sensing mechanism was investigated by a Job plot, ^1^H NMR titrations, and FT-IR spectra analysis, which demonstrated a chelation-enhanced fluorescence mechanism. Finally, a contact mode detection (test strips) between 10 and Hg^2+^ ions study shows a convenient and cost-effective strategy for naked-eye detection of Hg^2+^ ion. The electron-donor ability in the pyrazole moiety is a key property for the large changes in the photophysical behavior.

Magnetic core–shell nanoparticles as special immobilizing carriers of molecules for different applications have aroused great interest in current research because they are biocompatible, easily reusable, and stable against degradation.^90^ Among all reported nanocomposites, Fe_3_O_4_@SiO_2_ has the advantage of easy separation by an external magnetic field because of its magnetic core. Thus, probes attached to Fe_3_O_4_@SiO_2_ can be used both as sensing materials and for removing heavy metals from contaminated water. With this motivation, a novel pyrazole-derivative-functionalized Fe_3_O_4_@SiO_2_ fluorescent probe 11 was developed, and its selective Hg^2+^ detection properties were studied by Karimi and his research group (Fig. 7b).^91^ Upon the addition of Hg^2+^ ions, probe 11 shows a decrease in the fluorescence intensity band at 463 nm, when excited at 225 nm. A very low LOD was observed (7.6 nM) and the analysis can be done in water solutions. Moreover, this approach has the advantage that the probe can be easily separated from aqueous solution by an external magnet. Computational quantum chemistry methods revealed a nonparticipation in the coordination complex of the pyrazole core. Thus, in this approach, the pyrazole acts as a structural component and fluorophore. The proposed mechanism for this chemosensor was attributed to some well-known phenomena such as enhanced spin–orbit coupling or energy or electron transfer of Hg^2+^.

Motivated by the advantage of pyrene derivatives, that is, their ability to exhibit well-defined monomer and excimer emissions and consequent changes in emission spectral signatures, Goswami *et al.* reported a new pyrene–pyrazole-based rotamer chemosensor 12 for the highly selective detection of Hg^2+^ ions on the nanomolar scale (Fig. 7c).^92^ The addition of increasing amounts of Hg^2+^ ions led to a gradual decrease in the absorption intensity along with isosbestic points located at 315 nm and 340 nm. Moreover, as the concentration of Hg^2+^ increases, the emission band at 438 nm decreases due to 12–Hg^2+^ complex formation, as confirmed by ESI-MS and NMR experiments, and DFT calculations, disrupting the intermolecular pyrene–pyrene stacking of the excimer and, thereby quenching the emission intensity of 12. The chemosensor showed high selectivity toward other cations, and reversibility when Na_2_H_2_EDTA solutions were used, and the LOD of sensor 12 was determined to be 9.2 nM. The utility of 12 in live-cell imaging experiments was tested and establishing that 12 is able to detect cellular cytoplasmic Hg^2+^ in HepG2 cells. Importantly, in this probe, the pyrazole plays a significant role in Hg^2+^ sensing because its coordination point is crucial for the structural modification that induces the photophysical changes observed.

**Fig. 8:**
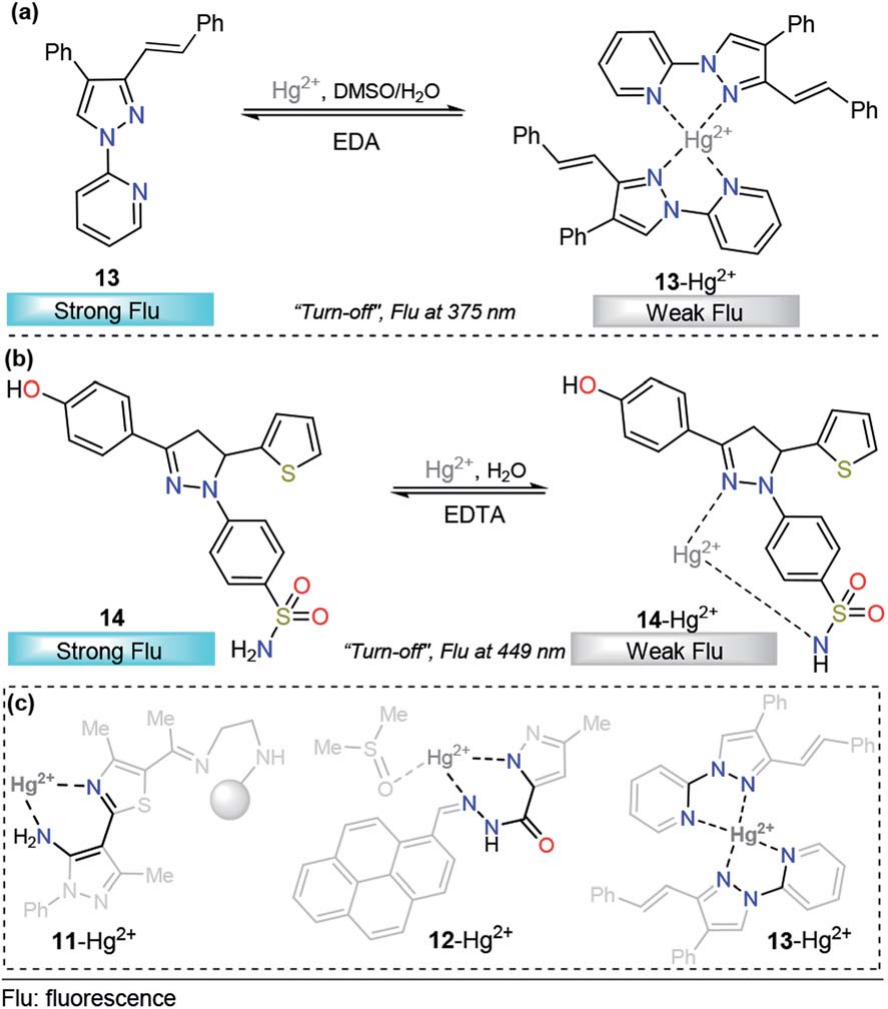
Probes for Hg^2+^ sensing based on pyrazoles (a) 13 and (b) 14. (c) Structural relationship between the pyrazole-based probe for Hg^2+^ sensing.

Taking advantage of our experience in pyrazole derivative synthesis, we designed and synthesized a new pyrazole derivative 13 for the detection and quantification of cations; this compound contains a fluorescent 1-(2-pyridyl)pyrazole unit, which also acts as a binding site due to the nitrogen positions (Fig. 8a).^93^ Compound 13 was evaluated as a fluorescent probe for cation detection since its structure has two donor nitrogen atoms at pyridine and pyrazole rings suitably located to achieve the formation of chelates. Upon the addition of different cations to 13 solutions in EtOH/H_2_O (9 : 1) negligible absorption changes were observed. However, the fluorescence band in the range of 350–400 (*λ*_exc_ = 320 nm) suffers a dramatic intensity decrease only when Hg^2+^ is added to the solution. The fluorescence quenching observed was attributed to the coordination of 13 with the Hg^2+^ ion in a 2 : 1 stoichiometry, which promotes a ligand-to-metal charge-transfer (LMCT) process in the excited state. This quenching shows a linear behavior suitable for analytical quantification of Hg^2+^ with very low LOD (0.31 μM) and reversibility upon ethylenediamine addition (Fig. 8a). However, further studies show changes in the fluorescence intensity when other cations were added to the solution, which did not allow us to selectively identify the chemosensor response in the presence of Fe^3+^, Co^2+^, Ni^2+^, and Cd^2+^. Despite the interference of some transition metals, pyrazole–pyridine derivatives can be incorporated in future design of this metal detection system due to the simplicity, available synthetic methods, high quantum fluorescence yields (66%) and suitably located N donor atoms to form chelates.

As we described in detail previously, pyrazolines have often been used for Zn^2+^ detection. However, with proper substitutions, pyrazolines can also be applied for sensing mercury. For example, Gul and Bozkurt reported an interesting study that use pyrazoline derivative 14 for Hg^2+^ sensing (Fig. 8b). In probe 14, the pyrazoline core is linked to 4-hydroxyphenyl group at position 3, a benzenosulfonamide group at position 1, and a thiophene-2-yl substituent at position 5. In the earliest photophysical studies, compound 14 exhibited an absorption band at 355 nm and relatively high-intensity fluorescence at 449 nm (*ϕ* = 0.62). Upon the addition of multiple metal ions, it was observed that these cations have no significant effect on the absorption spectrum of 14. However, the fluorescence spectrum indicated that the Hg^2+^ ion significantly decreased the fluorescence intensity (*ϕ* = 0.13) of ligand 14, while other metal ions had little or no significant effects on the fluorescence intensity of this ligand (*ϕ* = 0.66–0.49). This chemosensor showed a linear range from 20–200 μM, and a LOD of 14.54 μM was calculated. In accordance with the EDTA test, chemosensor binding to Hg^2+^ was found to be reversible, and reusability of the probe was proposed. Moreover, the test performed using tap water suggested that compound 14 could be used in real samples over a wide range of pH values. Finally, the authors used FTIR and NMR titrations to investigate the interaction between 14 and Hg^2+^, and they found that the hydroxyl group, the nitrogen atom in the pyrazoline ring and sulfonamide moiety are the main groups responsible for metal coordination.^94^

After a short analysis of the above-cited examples, we observed a tendency of Hg^2+^ ions to form stable 5- or 6-membered rings with the binding sites of the probes, except for the interaction proposed by H.-I. Gul in complex 14–Hg^2+^. This process could be a driving force for the selectivity of the different compounds, and pyrazole derivatives play an essential role in the formation of the stable rings (Fig. 8c).

#### 2.1.4. Aluminum(iii)

Aluminum (Al), the most abundant metal element in the Earth's crust, has been widely applied in industry and daily life.^95,96^ However, as a non-essential element for living systems, the frequent use of Al-containing products can lead to overloading of Al^3+^ in the living body, which may cause Al-related bone disease and various neurodegenerative diseases such as dialysis encephalopathy, amyotrophic lateral sclerosis, Alzheimer's disease, and Parkinson's disease.^97–99^ Hence, it is highly necessary to develop a sensitive approach for detecting and controlling Al^3+^ levels in environmental and biological appraisals.^100^

Imine- and hydrazide-type anchoring groups have been broadly used in the cation chemosensor area. The possibility of suffering photoisomerization of these groups, and consequently, the quenching of fluorescent states, open the door for a number of applications.^101,102^ In particular, Al^3+^-selective probes with an imine or hydrazide group, in which the coordination with Al^3+^ blocks the *cis*–*trans* isomerization and promotes a “*turn-on*” behavior of the fluorescence, have been extensively studied.^103–107^ Recently, pyrazole derivatives and hydrazide type groups have been incorporated into the design of high-performance probes for Al^3+^.

**Fig. 9:**
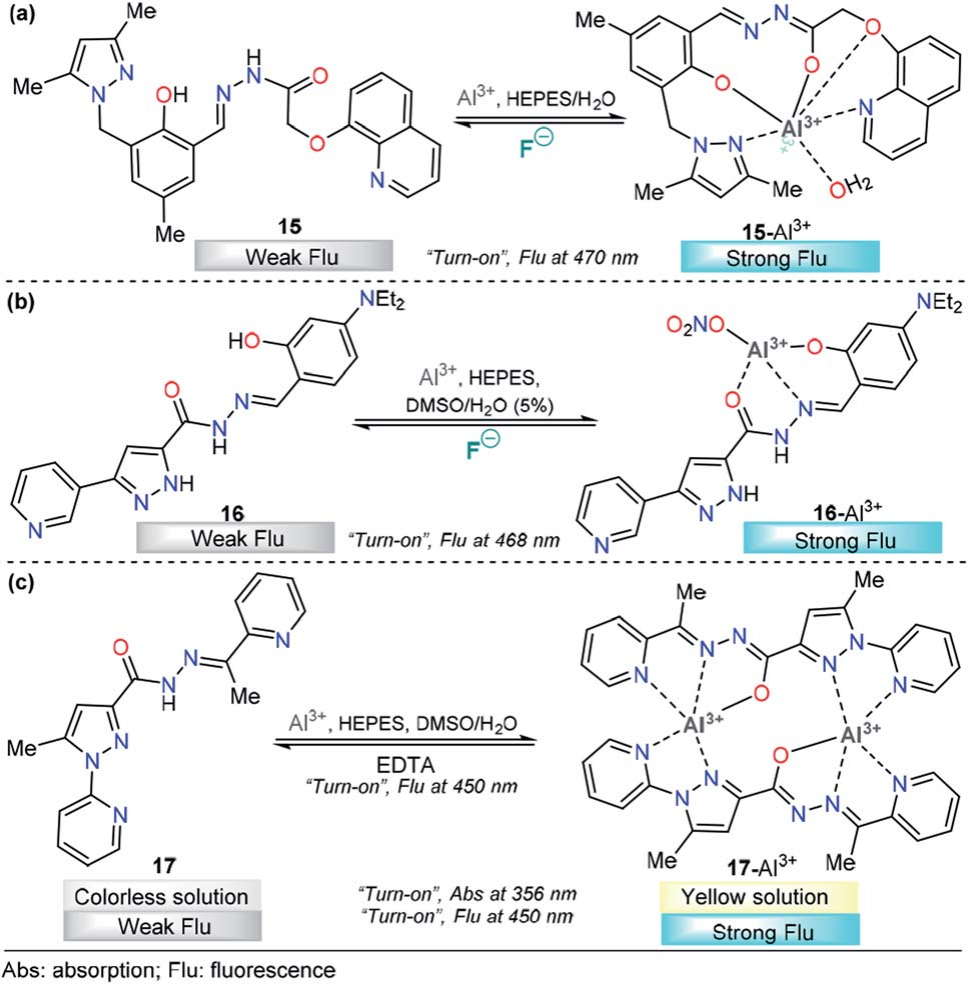
Pyrazole-based probe for Al^3+^ chemosensing. Three derivatives of (a) trialkylpyrazole 15, (b) 3-(3-pyridyl)pyrazole 16 and (c) *N*-(2-pyridyl)pyrazole 17 are shown.

For example, chemodosimeter 15 developed by Ali's group can selectively detect Al^3+^ in the presence of other related metal ions that exist in purely aqueous medium, 10 mM HEPES buffer at pH 7.2 (Fig. 9a). This probe is equipped with 8-hydroxyquinoline as the fluorescent unit and 3,5-dimethylpyrazole and a phenolic-hydrazide moiety as the binding site. Upon gradual addition of Al^3+^, there is a decrease in the absorbance at 330 nm along with the development of a new absorption band at 380 nm, and no significant change in the absorption with other tested metal cations. This result indicated a selective complexation between 15 and this cation. On the other hand, in the absence of Al^3+^ ions, 15 shows a very low-intensity band (*ϕ* = 0.002, *λ*_exc_ = 380 nm) owing to the combined effects of excited state intramolecular proton transfer (ESIT) from the phenolic –OH to azomethine –N and rotation around the C=N bound (*cis*–*trans* isomerization). However, upon addition of Al^3+^ to a solution of 15, an enhancement of the fluorescence intensity (*ϕ* = 0.002 → 0.28) was observed as a result of metal coordination, and then, both ESIT and rotational isomerization were efficiently blocked. Moreover, theoretical calculations support the formation of the 15–Al^3+^ complex. The LOD for this probe was found to be as low as 4.29 nM. The reversible binding of Al^3+^ to 15 was confirmed by reacting with excess F^−^ under both extra- and intracellular conditions using HepG2 cells as a biological sample. The pyrazole is a key coordination binding site in this chemosensor and allows the formation of the highly fluorescent complex 15–Al^3+^ and its quantification.^108^

Recently, Biang's group reported an acylhydrazone derivative containing pyridine–pyrazole as fluorescent chemosensor 16 for selective detection of Al^3+^ (Fig. 9b). The chemosensor displays a low-intensity fluorescence band at 468 nm when excited at 400 nm. However, upon the addition of Al^3+^, a fluorescence increase at 468 nm is observed, which can be ascribed to the formation of a complex between 16 and Al^3+^. The coordination mode of 16 with Al^3+^ was explored by ^1^H NMR spectroscopy and ESI-MS and supported by quantum chemical calculations. The analysis reveals nonparticipation of the pyrazole nitrogen atoms in the coordination with the metal. Thus, the pyrazole unit is used just as a fluorophore in this probe. In addition, the resultant 16–Al^3+^ complex could act as an “*on–off*” fluorescence chemosensor for F^−^. Probe 16 and complex 16–Al^3+^ have been applied to imaging in lysosomes of HeLa cells, in which both Al^3+^ and F^−^ ions can be observed at 30 μM and 20 μM, respectively. The LOD for Al^3+^ was as low as 62 nM, and the method could be performed in water as the solvent. Finally, the authors demonstrated an aggregation-induced emission enhancement (AIEE) phenomenon by using time-resolved fluorescence, dynamic light scattering (DLS) and transmission electron microscopy techniques.^109^

A new pyridine–pyrazole based chemosensor 17 was synthesized through a very simple method by Goswami and his research group and applied to sensing various cations (Fig. 9c). Probe 17 shows characteristic absorption spectra with an absorption band at 380 nm and displays a low-intensity fluorescence band at 450 nm (*ϕ* = 0.051, *λ*_exc_ = 300 nm). Probe 17 effectively and selectively recognized Al^3+^ ions in the presence of other cations in 2 : 8 DMSO/H_2_O in HEPES buffer (pH = 7.4) with both colorimetric and fluorescent techniques. The incremental addition of Al^3+^ causes a decrease in the band intensity at 280 nm, and a new band appears at 356 nm, with a clear isosbestic point at 325 nm. Moreover, a “*turn-on*” behavior (*ϕ* up to 0.610) was observed, in the emission band at 450 nm upon addition of Al^3+^ to the solution of probe 17. The LOD was calculated to be 1.2 nM, and the reversibility of the chemical reaction between 17 and Al^3+^ was demonstrated by using EDTA solution. The authors also constructed a paper-based fluorescent chemosensor of 17 for naked-eye detection of Al^3+^, with excellent results. Computational calculations reveal the formation of a dimeric structure between 17 and Al^3+^, in which the pyrazole plays an important role in the metal coordination and then the chelation-induced enhanced fluorescence effect, which suppress the PET process. Considering the selectivity of 17 toward Al^3+^, it was further assessed for its Al^3+^ sensing ability in living cells such as HepG2. A concentration as low as 4 μM was observed for 80% cell viability, and intense intracellular fluorescence was observed due to the formation of complex 17–Al^3+^. Hence, ligand 17 with low cytotoxicity and biocompatibility can be used for cellular cytoplasmic Al^3+^ ion detection in biological samples.^110^

**Fig. 10:**
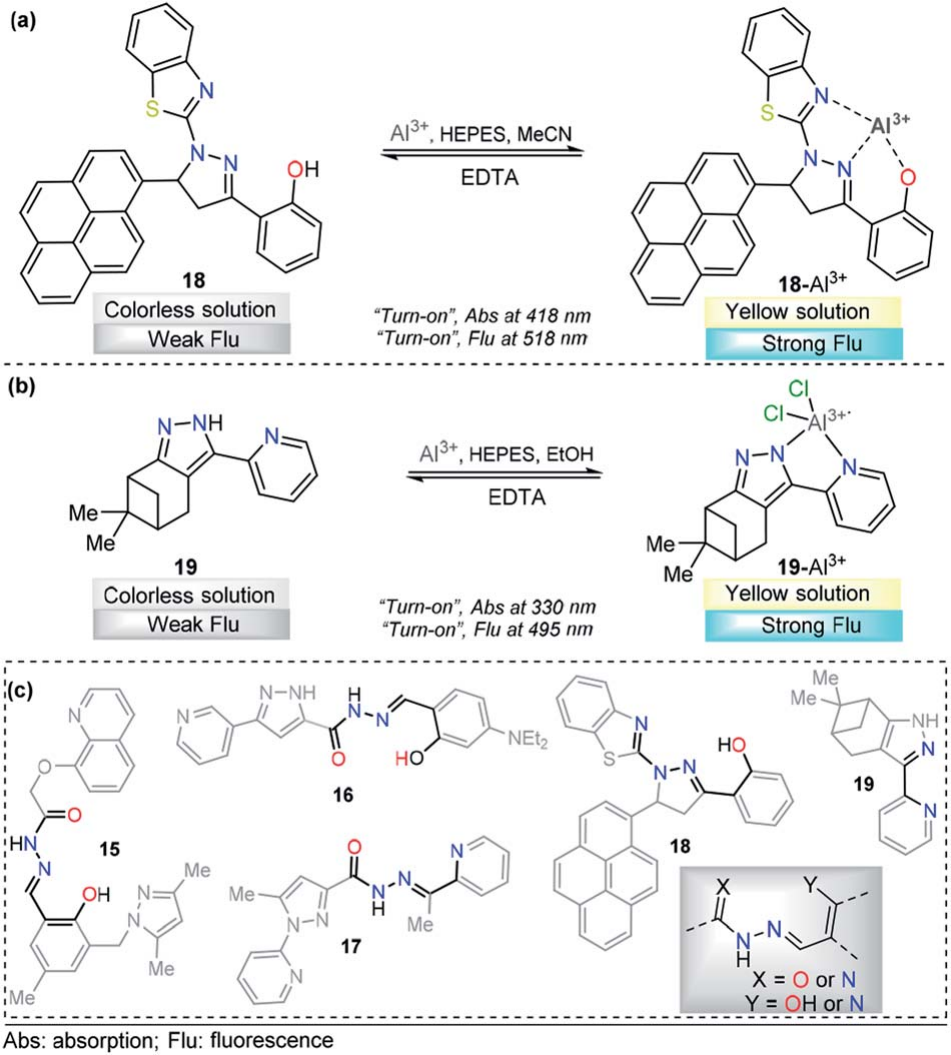
Probes for Al^3+^ sensing based on pyrazoles (a) 18 and (b) 19. (c) Structural similarity between compounds 15–19.

A pyrene-appended pyrazoline 18 was designed and synthesized by P. Kannan and R. Manjunath, and its colorimetric and fluorescence applications for aluminum sensing were studied (Fig. 10a). In the absence of various metal ions and using HEPES–MeCN–water (pH = 7.2, 50% v/v) as the solvent, compound 18 exhibited a sharp absorption band at 344 nm, and no characteristic absorption appeared at approximately 418 nm. Upon the addition of multiple ions to 18, Al^3+^ alone yielded a new absorption band centered at 418 nm. The intensity of this band gradually increased with increasing Al^3+^ concentration, corresponding to a colorimetric change (from colorless to green). Probe 18 exhibited no significant fluorescent emission at 518 nm (*ϕ* = 0.047). When the concentration of Al^3+^ increased, a new emission peak appeared at 518 nm, with a high quantum yield (*ϕ* = 0.574). Reversibility experiments revealed that the nature of the binding interaction between 18 and Al^3+^ is reversible. Unfortunately, the authors did not show any analytical studies related to the linear range or limit of detection. Then, compound 18 was proposed as a potential chemosensor for aluminum in a wide range of pH values (7–13). By synthesizing an analog, the authors showed that the hydroxyl group is essential for metal coordination, and taking into account the 1 : 1 ligand–metal stoichiometry, from Job and Benesi–Hildebrand plots, a binding mode of 18–Al^3+^ was proposed, in which the nitrogen atom in the pyrazoline plays a key role. In addition, fluorescence imaging experiments of Al^3+^ ions in living cells (mouse embryonic fibroblast (3T3-L1) cells) demonstrate the value of this probe in practical applications in biological systems.^111^

**Fig. 11:**
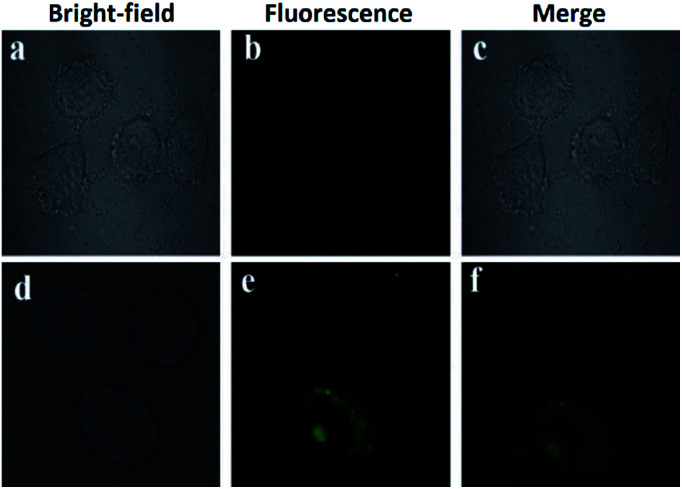
(a) Fluorescent image of HeLa cells treated with probe 19 (5.0 μM) in the absence of Al^3+^; (b) microscope image of HeLa cells treated with probe 2a (5.0 μM) in the absence of Al^3+^; (c) merged image of frames (a) and (b); (d) microscope image of HeLa cells treated with Al^3+^ (5.0 μM) and probe 19 (5.0 μM); (e) fluorescence image of HeLa cells treated with Al^3+^ (50.0 μM) and probe 19 (5.0 μM); (f) merged image of frames (d) and (e). Reprinted with permission from ref. 112 of RSC.

A different approach was used by Wang *et al.* incorporating a β-pinene-pyrazole derivative, compound 19 (Fig. 10b). The authors combined the aforementioned fused pyrazole with a pyridyl group at position 5 and found outstanding properties for aluminum sensing. The absorption spectrum of 19 was characterized by an absorption band at 280 nm. Only upon the addition of Al^3+^ (10 equiv.) to a solution of probe 19, did the absorption peak at 280 nm almost disappear, and the peak at 330 nm was greatly enhanced. The probe 19 solution in EtOH/HEPES buffer (pH = 7.4) was essentially nonfluorescent when excited at 330 nm. After the addition of Al^3+^ to the solution of compound 19, green fluorescence dramatically appeared (*λ*_em_ = 495 nm, *ϕ* = 0.49), and the fluorescence intensity increased linearly between the fluorescence intensity and the low Al^3+^ concentration in the range 0–12.0 μM; the LOD for Al^3+^ was found to be 81 nM. By using HRMS, NMR, and Job plot experiments as well as theoretical calculations, the authors proposed a coordination mechanism that explains the observed photophysical properties. In this binding mode, pyrazole and pyridine coordinated an aluminum atom and an ICT process took place, inducing pronounced fluorescence enhancement. By using EDTA solutions, the reversibility of the binding 19–Al^3+^ was confirmed. Novel probe 19 was used for Al^3+^ detection in water (tap, distilled and lake) and food samples (chips, fried chicken, sausage, tea, biscuit, and baby biscuit) with good recovery, ranging from 96 to 103%. Therefore, this compound can be applied as part of a simple method to detect the concentration of Al^3+^ in various water and food samples and in a wide pH range (1 to 9). Additionally, probe 19 was successfully utilized to label intracellular Al^3+^, indicating its promising applications in living cells (Fig. 11).^112^

Although the compounds reviewed for Al^3+^ sensing have different chromophores/fluorophores, they share the structural component responsible for metal coordination (Fig. 10c). Even compound 19 has a fragment of that structural component. These observations could allow a precise design of probes for this metal.

### 2.2. Other cations

Pyrazole derivatives have been used for the monitoring of other important metal ions such as Fe^3+^, Cr^3+^, and Ag^+^. The quantification of these metals finds a broad number of applications in human health,^113^ industries,^114^ agriculture, and environmental purposes.^115^ Thus, the development of reliable, sensitive and selective methods for their detection is of extensive importance.^116–118^ Consequently, below we present some representative pyrazole derivatives that have shown outstanding properties in the area.

#### 2.2.1. Iron(iii)

**Fig. 12:**
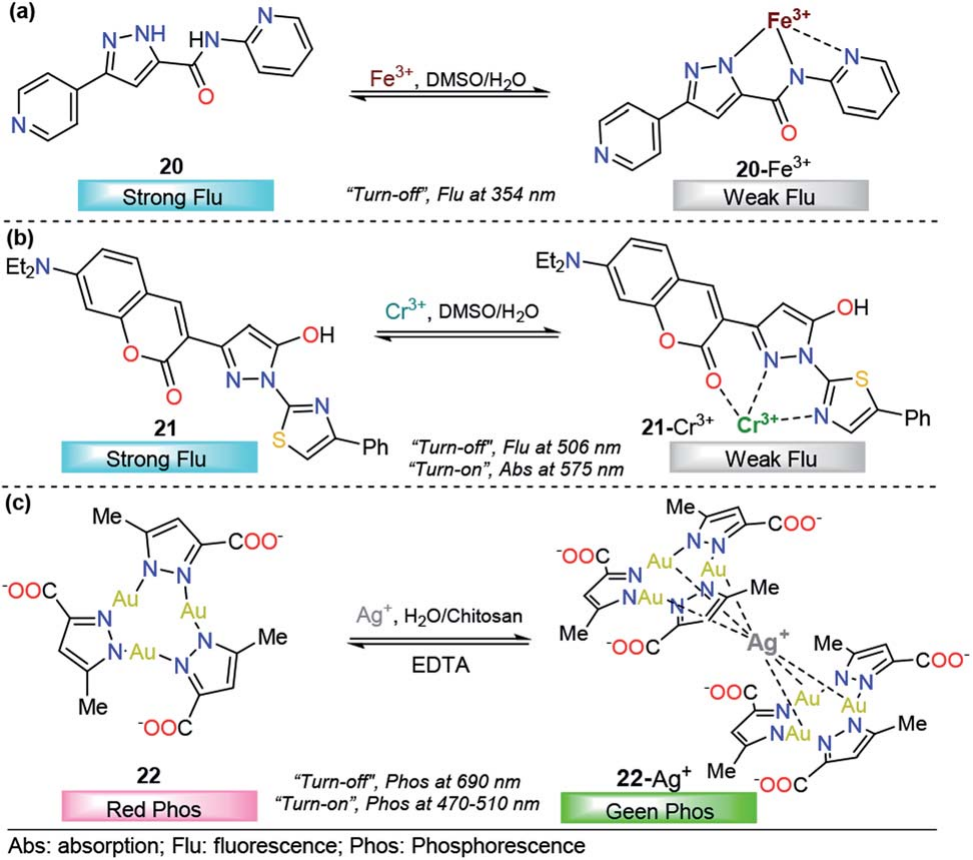
Probes for Fe^3+^, Cr^3+^ and Ag^+^ based on pyrazoles (a) 20, (b) 21 and (c) 22.

Madhu *et al.* designed and developed a very simple pyridine–pyrazole-based dye 20 as a dual colorimetric and fluorescent chemodosimeter for Fe^3+^ ions (Fig. 12a).^119^ After the addition of Fe^3+^ ions, in a 9 : 1 DMSO/H_2_O solution of 20, a new absorption band was observed at 340 nm; however, Fe^2+^ also produced some small interaction with the probe 20. This means that the chemosensor cannot differentiate between Fe^3+^ and Fe^2+^ through a colorimetric technique. A linear relationship was observed and a very low LOD (57 nM) was found. On the other hand, upon increasing the concentration of Fe^3+^ the emission band at 350 nm (*λ*_exc_ = 310 nm) in 9 : 1 DMSO/H_2_O decreases linearly in intensity, with a LOD of 88 nM. The sensing process of Fe^3+^ ions is due to the tricoordinated 20–Fe^3+^ complex, and the dramatic changes in the fluorescence spectra can be attributed to the highly paramagnetic nature of the metal. This assumption was validated by replacing the 2-pyridyl moiety by phenyl ring in a 20-analog. In this analog, the quenching of the fluorescence intensity is less pronounced when compared with the probe 20. Furthermore, DFT calculations confirms the complexations between 20 and Fe^3+^, and a contribution for fluorescence quenching *via* inhibition of the ICT process was proposed. Nevertheless, the small quenching in the fluorescence band caused by Fe^2+^ indicates a negligible contribution of the ICT mechanism in the detection process. Based on its highly nucleophilic N atom and outstanding photophysical properties, when joined to pyridine moiety, pyrazole plays an important role in both structural and photophysical properties of the probe 20.

#### 2.2.2. Chromium(iii)

Regarding a chromium chemodosimeter, S. P. Rajendran *et al.* designed and synthesized a novel dye containing coumarin–pyrazole derivatives, 21, and studied its sensing properties toward cations (Fig. 12b).^120^ Before the addition of metal ions, probe 21 exhibits a broad absorbance maximum at 446 nm in DMSO. Upon addition of Cr^3+^ to 21, the absorption peak blueshifted from 446 to 409 nm, a new band appeared at 575 nm, and the color of the solution also changed from fluorescent green to yellow. Moreover, in the absence of Cr^3+^ ions, the fluorescent spectrum of 21 exhibited a strong green emission peak at 506 nm (*λ*_exc_ = 446 nm). The sequential addition of Cr^3+^ ions from 0 to 100 nM to the solution of 21 showed a gradual decrease in the intensity of this emission band, with a good linear relationship in the concentration range from 5 to 75 nM, and the LOD was estimated to be 37 pM. These absorption and emission spectra changes were attributable to the coordination of Cr^3+^ with ligand 21. Additionally, quantum chemical calculations confirmed the LMCT mechanism for the observed fluorescence quenching, and time-resolved fluorescence techniques supported the formation of a nonfluorescent complex *via* static quenching. The pyrazole moiety plays an essential role in complex formation with Cr^3+^ ions, acting as a coordination point and allowing colorimetric and fluorescence detection of this cation. The extended wavelength sharp fluorescence emission of the 21 sample was successfully employed in the intracellular recognition of Cr^3+^ in living A-549 cells.

#### 2.2.3. Silver(i)

As is well known, compared to fluorescent complexes, phosphorescent complexes have a plethora of unique and advantageous photophysical properties such as higher quantum yields, longer lifetimes, larger Stokes shifts, and higher sensitivity and/or selectivity to local environments.^121^ Based on these benefits, Upadhyay *et al.* recently reported a phosphorescent chemosensor for silver ions (Ag^+^) based on a trinuclear Au^+^ pyrazole complex 22 stabilized in aqueous chitosan polymer media (Fig. 12c).^122^ In this case, the pyrazole acts as a coordination point for the gold trinuclear complex through the two nitrogen atoms. The phosphorescent spectrum of 22 was obtained (*λ*_exc_ = 290 nm) with chitosan in deionized water, and a red emission band at 690 nm was observed. The addition of Ag^+^ ions into aqueous solution of 22 results in a photoluminescence color change from red to green, which is coupled with the simultaneous growth of a new peak at approximately 470–510 nm (*λ*_exc_ = 325 nm), and the proposed mechanism for silver sensing was the formation of an adduct such as 22–Ag^+^ (Fig. 12c). The sensing process using 22 is simple and fast, and the changes can even be detected by the naked eye, using water as the solvent and exhibiting LODs as low as 5 ppb. Moreover, 22 is able to differentiate between Ag^+^ and silver nanoparticles (AgNPs), which is a great utility to help nanoparticle research differentiate and understand whether the toxicity of AgNPs is due to AgNPs alone, leaching of silver ions, or a combination of both.^123^ Finally, the complex was proposed for contaminated water remediation without any interferences.

### 2.3. Anions

Anion sensing has grown to be a major field in recent years, with particular emphasis on fluoride (F^−^) and CN^−^ detection due to their physiological and environmental significance. Sensing of F^−^ ions is a relevant research area^124^ because of the functional diversity on the anion, which is both beneficial and detrimental. For example, it is well established that fluoride ions play an important role in preventing dental caries and treating of osteoporosis,^125^ while excess fluoride ions causes several adverse effects such as fluorosis, kidney disorder,^126^ and bone^127^ and skeletal cancer.^128^ On the other hand, salts containing CN^−^ are among the most dangerous toxins to living organisms. Cyanide acts as an inhibitor of mitochondrial cytochrome c oxidase and thus disrupts the electron transport chain, leading to eventual blockage of cellular respiration.^129,130^ Recently, a number of pyrazole-based probes have been designed and studied for F^−^ and CN^−^ quantification, and outstanding results have been achieved.

#### 2.3.1. Fluoride

Using probes for the detection of fluoride ions in living cells and the environment through colorimetric and/or fluorescence techniques is particularly important and has attracted many chemists' attention.^131^ Fluorine has the highest electro-negativity and can form strong hydrogen bonds or deprotonated species with NH and OH. When F^−^ ions form hydrogen bonds with these probes, the physical properties of these compounds are displayed by color or fluorescent signal changes.^132^ By taking advantage of this property, pyrazole derivatives have been successfully used as key moieties in a number of chemosensors with good performance. In particular, the pyrazole fragment has been equipped with groups with proton-donor potential that, after acid-based reaction with F^−^, change the photophysical properties.

**Fig. 13:**
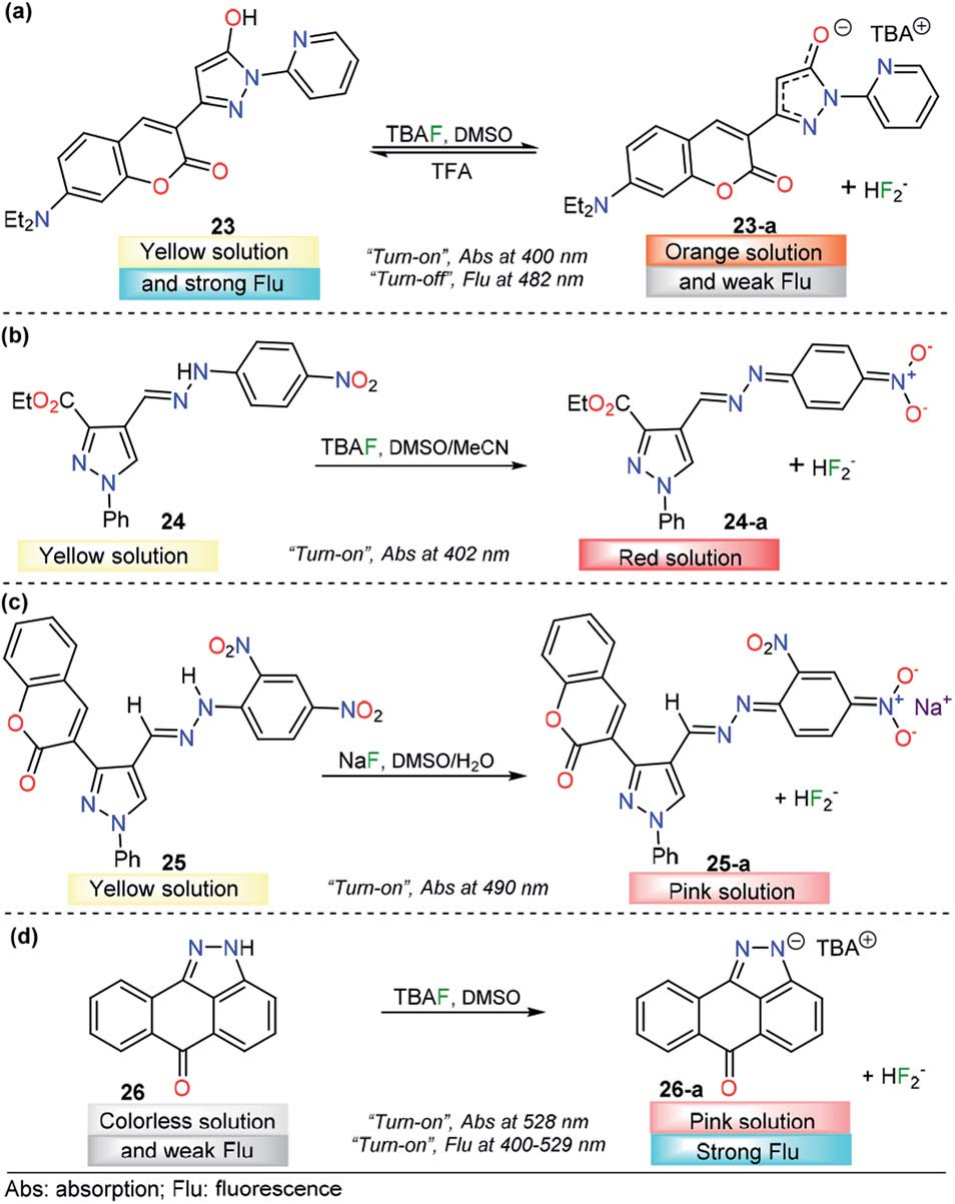
Probes for F^−^ ions based on the pyrazole derivatives (a) 23, (b) 24, (c) 25, and (d) 26.

Seferoğlu and his research team reported an integrated coumarin–pyrazole anion probe for sensing organic F^−^ (tetrabutylammonium salt, TBA), compound 23 (Fig. 13a). This probe was equipped with a coumarin group as the signaling unit (fluorophore/chromophore); a pyrazole at the 3-position of the coumarin ring partly tunes the luminescent properties of the coumarin core, an OH group acts as an H-bond donor, and a pyridine moiety is placed at position 1 in the pyrazole. A solution of 23 in DMSO showed an absorption band at 400 nm and an emission maximum band at 482 nm (*λ*_exc_ = 400 nm). Upon the addition of F^−^, the absorption and emission bands of 23 change dramatically. The former was shifted hypsochromically, and a new band at 401 nm was created. Meanwhile, the intensity of the emission band gradually decreases in a “*turn-off*” fashion. In the presence of other anions, chemosensor 23 did not show significant changes in either the absorption or emission spectra. The nature of the interaction between 23 and F^−^ ions was established by NMR experiments. The photophysical response observed was attributed to deprotonation of 23 upon interaction of F^−^ with the –OH group and the formation of the FHF^−^ anion (*δ*: 16.15 ppm, *J*_HF_ = 121 Hz). Theoretical explanations at the DFT level indicated an excited-state intramolecular proton transfer mechanism. Moreover, with TFA addition, the emission intensity of 23 in CH_2_Cl_2_ significantly increased, which can be observable even by the naked eye. Thus, 23 can also detect pH changes visually or under a portable UV lamp by fluorescence change. However, further analytical information is needed in order to evaluate the potential applications of this compound.^133^

The use of a hydrazone group as a proton source is a recurrent approach in the fluoride ion-sensing field. The combination of stabilizing and/or chromogenic groups has been the design principle of a variety of probes with stupendous results.^134–136^ Shirvastava *et al.* reported a pyrazole-4-dinitrobenzene linked by a hydrazone bridge based colorimetric fluoride chemodosimeter 24 as a simple and efficient semiquantitively chemosensor (Fig. 13b). This probe exhibited a significant visual color change from yellow to red and showed selectivity toward F^−^ ions in DMSO/MeCN (1 : 9 v/v) medium. In the absence of any anion, chemosensor 24 displayed an absorption band at 402 nm in DMSO/MeCN (1 : 9 v/v) medium. Upon gradual addition of a standard solution of tetrabutylammonium fluoride, the intensity of the absorption band at 402 nm progressively decreased, with a simultaneous increase in the intensity of a new band at 557 nm. Under the same conditions, no significant changes were observed with other common anions. ^1^H NMR titration experiments revealed that probe 24 showed selective sensing of fluoride ions *via* hydrogen bonding interactions in the presence to other ions. The authors also demonstrated the importance of the structural composition in the F^−^ selectivity.^133^

A similar approach was conducted by Agarwal *et al.*, who described a low-molecular-weight selective probe for F^−^ detection, compound 25 (Fig. 13c). In this case, the pyrazole unit is attached to a 2,4-dinitrobenzene group through a hydrazone linkage that acts as the recognition site. Moreover, a coumarin moiety was attached at position 3 of the pyrazole. In the absence of an external analyte, UV-vis spectrum of receptor 25 is characterized by a maximum at 410 nm in DMSO. The absorption band of 25 at 410 nm gradually decreases only upon the addition of the fluoride anion, and a new peak appears at 490 nm, with a color changes from yellow to pink. The chemodosimeter can detect F^−^ at concentrations as low as 0.95 ppm, and the NMR and FTIR analyses indicated that an acid–base reaction occurs between 25 and F^−^ to produce the conjugated base 25. The authors proposed a plausible explanation based on the resonance stabilization of the conjugated based of 25, after a chemical reaction with fluoride ions. The probe was proposed as a simple and selective semi qualitative naked-eye chemosensor for fluoride.^137^

Fused-pyrazole derivatives have also been tested for fluoride recognition, and an astonishing example was reported by Hu and his team in which a simple anthra[1,9-*cd*]pyrazol-6(2*H*)-one chemodosimeter 26 was designed for the highly selective and sensitive detection of F^−^ ions (Fig. 13d). Probe 26 exhibited a major absorption band at 400 nm and a fluorescence band at 529 nm (*ϕ* = 0.03, *λ*_exc_ = 420 nm). For colorimetric and fluorimetric detection of F^−^ ions by using chemosensor 26, titration experiments were carried out in DMSO. Upon the addition of a fluoride solution into a solution of probe 26, the color of the yellow sensor faded (colorless), accompanied by an absorbance change at 440 nm, which gradually decreased and eventually disappeared after the addition of 7.8 equivalents of CN^−^ ions. When the F^−^ ion was added, it was found that a new absorption band appeared at 528 nm, while the intensity of the absorption band at 400 nm decreased correspondingly. On the other hand, upon treatment of 26 with fluoride ions, a dramatic increase in fluorescence was observed (*ϕ* = 0.37). The LOD for F^−^ using fluorescence intensity changes was estimated to be 0.28 μM. Among the anions tested, AcO^−^ and H_2_PO_4_^−^ only led to slight fluorescence changes in 26, which clearly indicated that this probe could act as a highly selective fluorescent sensor for fluoride ions over other anions. ^1^H NMR analysis and DFT calculations showed that the F^−^-induced colorimetric and fluorometric responses of 26 are simply driven by the deprotonation process of the N–H group in the pyrazole group.^138^ To the best of our knowledge, this result is the best for a pyrazole derivative probe in the F^−^ detection area.

#### 2.3.2. Cyanide

Among different anions, CN^−^ ions are extremely toxic and dangerous pollutants. Consequently, there is considerable interest in the colorimetric and fluorescent detection of CN^−^ ions at trace levels by using simple methods. The reaction-based probes that take advantage of the unique nucleophilic reactivity of CN^−^ display more specific selectivity and higher sensitivity. In general, the design strategy of the reaction-based probes for CN^−^ involves the nucleophilic addition reaction of CN^−^ to electrophilic C=C (conjugated with electron-withdrawing groups), C=O, C=N, or C=N^+^ double bonds. After the addition reaction, the π-conjugation of the probes is interrupted, which results in an obvious optical response.^4,139^ For this purpose, the pyrazole core has been combined with anysyl, pyridyl, or triphenylamine or fused with other heterocyclic moieties and equipped with highly electrophilic groups for CN^−^ sensing through nucleophilic addition-based mechanisms.

**Fig. 14:**
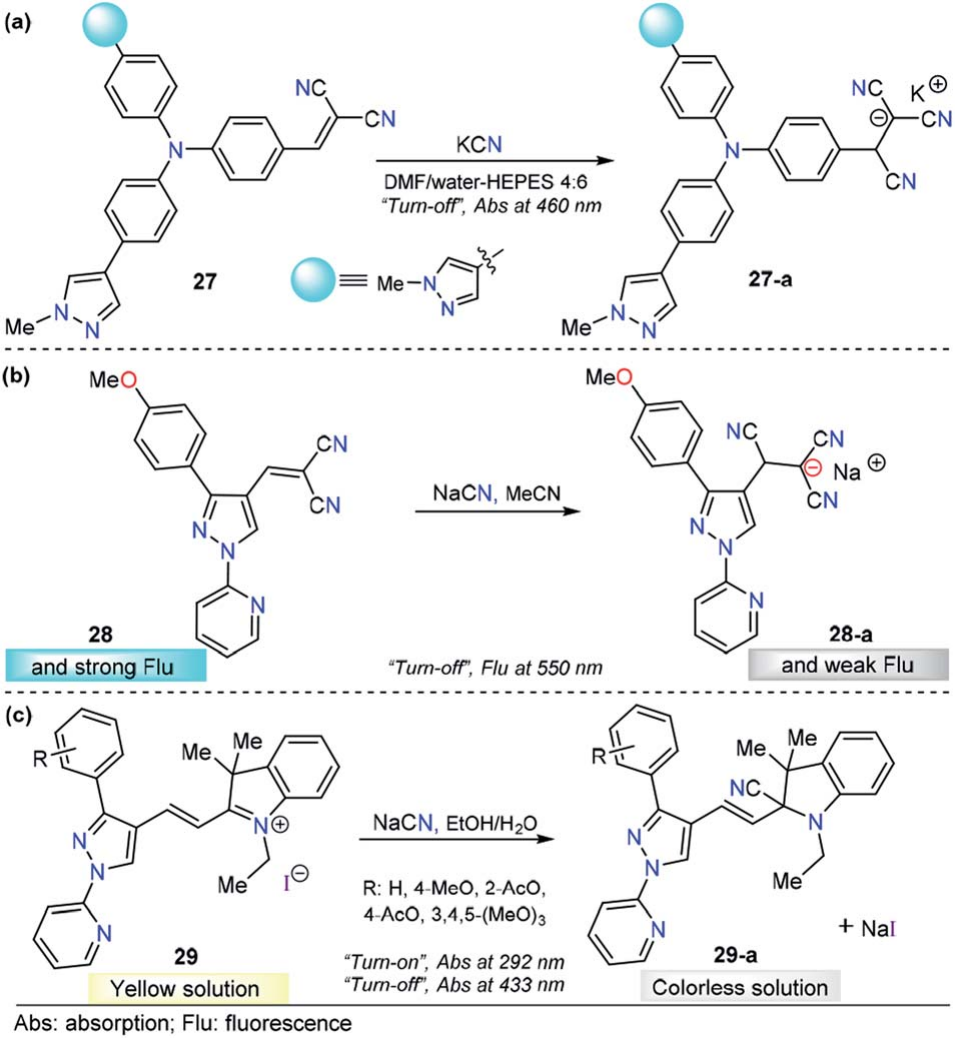
Pyrazole-based probe for CN^−^ chemosensing. Three derivatives of (a) *N*-methylpyrazole 27, (b) 4-dicyanovinylpyrazole 28 and (c) *N*-(2-pyridyl)pyrazole 29 are shown.

Siva and Beneto studied pyrazole–triphenylamine derivative 27 for CN^−^ sensing (Fig. 14a).^140^ Compounds 27 shows an absorption spectrum characterized by two absorption bands at 310 and 460 nm in 4 : 6 DMF/water–HEPES. Various anions were added to a solution of 27, and no significant change was found. When adding 1.0 equivalent of CN^−^ ions, the peak at 460 nm decreased in a linear way, and the LOD of 27 was found to be 44 nM. When compound 27 was excited at 320 nm, a moderate intensity emission band was observed at 400 nm. Upon the addition of CN^−^ ions, similar behavior was noted. This time, an increase in the band intensity was observed. However, no further experiments were carried out to quantify CN^−^ ions by fluorescence measurements. A mechanism in which the fluorophore switched its photophysical properties as a result of disrupted conjugation was proposed and supported by DFT calculations. By comparing 27 with similar triphenylamine derivatives, it can be noticed that introducing a pyrazole moiety causes a bathochromic shift (440 → 460 nm) in the absorption band. Triphenylamine–malononitrile probes have exhibited an emission band at around 580 nm in highly polar media.^141^ Notably, the presence of pyrazole in this probe shifted the emission band to 400 nm. Thus, this heterocyclic moiety plays an important role in both the ground and excited states of the probe. On the other hand, a similar performance was observed for chemodosimeter 27 when compared with a compound in which pyridine was attached to the triphenylamine–malononitrile probe. Compound 27 has low limits of detection and solubility in highly aqueous media.^142^

As part of our continuous efforts to develop new and efficient probes for detecting CN^−^ ions, we have developed pyrazole probes with interesting properties in this area. First, based on the interesting photophysical properties found in previous reports,^93,110,119,133,143,144^ a 1-(2-pyridyl)pyrazole group was used as the fluorophore and attached to the dicyanovinylene group as the recognition site, yielding chemosensor 28 (Fig. 14b).^145^ This compound displayed an absorption band at 360 nm in acetonitrile. The fluorescence spectrum showed an excitation wavelength of 360 nm in which an emission band appeared at 550 nm. Titration experiments in both absorption and emission techniques with CN^−^ solutions and other ions show that 28 is sensitive (LOD = 6.8 μM) and selective toward CN^−^ ions. HRMS (ESI) and ^1^H NMR titration revealed that a conjugated addition of CN^−^ into dicyanovinylene group of 28 takes place, and computational calculations support the spectral results by comparing the frontier orbitals in 28 and 28-a, demonstrating that charge transfer is interrupted in the adduct 28-a. Test strips were prepared with 28, and the minimal detectable concentration of CN^−^, by means of visual determination was 10 mM. However, the minimal concentration allowed in drinking water by the WHO is 1.9 μM, which is much lower than the LOD of 28. Moreover, due to the low polarity of the probe, the analysis needs to be done in at least 30% organic solvents. Consequently, the application of this compound is quite limited.

In view of the performance of probe 28 and taking into account its disadvantages, we envisioned that introducing a hemicyanine acceptor group will improve the photophysical properties and solubility in greener solvents such as water or ethanol (Fig. 14c). Therefore, we designed and synthesized a chemodosimeter group 29a–d by replacing the dicyanovinylene group in compound 28 with a hemicyanine salt; for a fair comparison, compound 29a with a CH_3_O group was selected. This compound displayed an absorption spectrum with absorption bands at 292 and 433 nm in 1 : 1 EtOH/H_2_O solution. Regarding the fluorescence experiments of 29a, the results were not significant in any of the evaluated solvents, as all compounds showed low quantum yields. Therefore, only UV-vis titrations were performed. Upon the addition of a number of anions, only CN^−^ caused a dramatic change in the absorption bands, with the disappearance of the ICT band at 433 nm and an increase in the intensity at 292 nm. These absorption bands demonstrated ratiometric behavior, analytical experiments revealed reaction times as low as 10 min, and the LOD was found to be 1.15 μM. In comparison with 28, chemodosimeter 29a has some advantages. First, excellent performance was achieved in greener solvents such as ethanol/water solutions.^146^ Second, the absorption band of 29a has a bathochromic shift of 73 nm. Third, a double channel (*λ*_292_ and *λ*_433_) can be used for anion sensing. Fourth, the LOD is much lower than the WHO permit in drinking water (29a: 1.15 μM, allowed 1.9 μM). However, probe 29a has poor fluorescence properties, and thus, this pyrazole can be used only as a colorimetric probe.

**Fig. 15:**
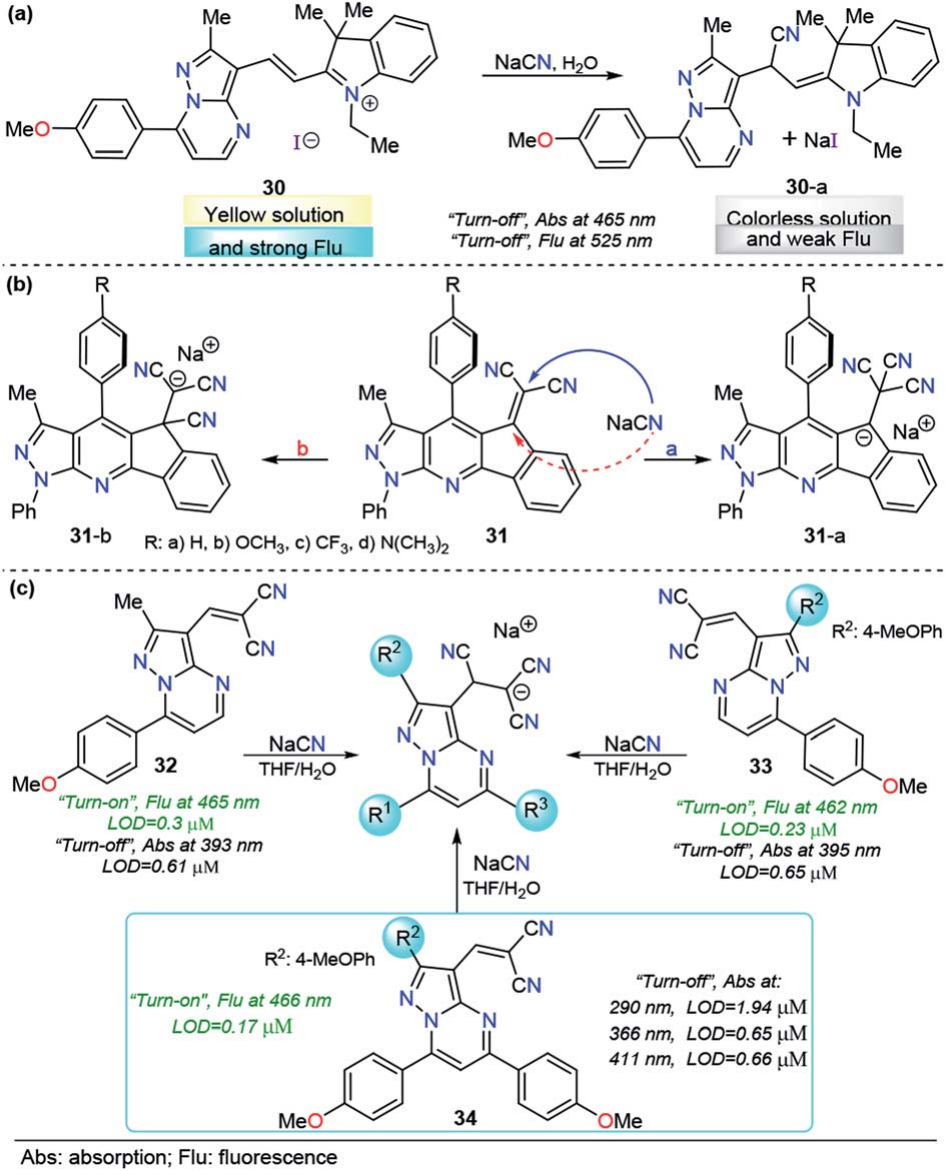
Probes for CN^−^ ions based on the fused pyrazoles (a) 30, (b) 31 and (c) 32–34.

As mentioned before, when compared with a single pyrazole analog, fused pyrazoles display a number of improved photophysical properties. This improvement is the result of extended π-conjugation and planarity and the introduction of additional electron-rich/poor atoms. Recently, we developed a simple, easy and one-pot approach^147^ to access functionalized pyrazolo[1,5-*a*]pyrimidines, a fused analogous of the 1-(2-pyridyl)pyrazole group, which demonstrated intrinsic outstanding fluorescent properties.^148^ Considering the aforementioned aspects together with our interest in developing efficient probes for toxic species such as cyanide, we used the pyrazolo[1,5-*a*]pyrimidine core as a chromophore/fluorophore in combination with hemicyanine to develop a new chemosensor for CN^−^ ions, compound 30 (Fig. 15a).^149^ The UV-vis spectrum of 30 in 100% water exhibited a charge transfer band at 465 nm with a high absorptivity coefficient (*ε* = 63 666 M^−1^ cm^−1^). When 30 was excited at 465 nm, it exhibited an intense fluorescence band at 530 nm (*ϕ* = 0.43). A complete study of probe 30 reveals a significant selectivity in the presence of other ions and a high sensitivity with both absorption and fluorescent techniques, with LODs as low as 0.6 and 0.086 μM, respectively. The regiochemistry of the reaction between the chemodosimeter 30 and CN^−^ ions was demonstrated by synthesizing compound 30–CN and characterizing it through ^1^H NMR and HRMS techniques. The high solubility in water and the dual (colorimetric and fluorimetric) response properties of 30 indicated that this fused pyrazole displayed improved behavior for CN^−^ sensing when compared with 28 and 29a.

The interesting results obtained with fused pyrazoles have pushed our research interest toward fused heterocycles for the design of molecules in the CN^−^ chemosensor field, and very recently, we reported a new group of pyrazolo-fused 4-azafluorenes, compounds 31 (Fig. 15b).^150^ The photophysical and computational studies of 31 showed that these products are modulable ICT fluorophores, and preliminary tests revealed that these compounds could be used as fluorescent chemosensors for CN^−^ detection in both “*turn-on*” or “*turn-off*” ways. In this case, the regioisomeric addition products 31-i or 31-ii could be obtained based on their better stabilization. The study of the design of chemosensors based on the structures of 31 is still ongoing.

Although reaction-based probes display good performance in sensing CN^−^ in environmental samples, when sensing endogenous CN^−^ in a biological sample, the probes still confront potential interference from some biological nucleophiles, such as biological thiols and hydrogen sulfide. These biological nucleophiles could attack the electrophilic double bonds and destroy the π-conjugation of a fluorophore similar to the action of CN^−^.^151^ Despite many efforts to develop probes for CN^−^ detection, to the best of our knowledge, no attempts have approached this interference disadvantage from the structural point of view.

Motivated by the aforementioned probe limitations, we recently studied the influence of introducing multiple electron-rich groups in the probe. The purpose was to increase the electron density in the electrophilic carbon of the dicyanovinylene group. This chemical modification will have an impact on the reactivity, allowing the reaction of the probe only with highly nucleophilic species. We selected as a fluorophore the pyrazole derivative with the best performance to date (the pyrazolo[1,5-*a*]pyrimidine core, probe 30) with one (32), two (33) and three (34) 4-methoxyphenyl (4-anisyl) groups as electron donors in their periphery.^152^ In this probe optimization, we found that the number of electron-donor groups and their position have a remarkable influence on the selectivity, kinetics, sensibility, and even on the number of channels of detection (Fig. 15c). The absorption band wavelengths of these probes, assigned to the ICT processes, range from 393 to 411 nm. Regarding the emission spectra, the fluorescence bands of these groups of probes are very close when excited at 300 nm. Interestingly, all three probes show dual CN^−^ chemosensing with LODs as low as 0.61 μM for probe 32 in absorbance measurements and 0.17 μM for probe 34 in fluorescence determination. More importantly, probe 34 displayed interesting advantages over the other probes in the study: it showed up to four wavelengths for detection channels, three of them substantially below the World Health Organization limits for drinking water; second, it reached the best selectivity in the group, even in the presence of high concentrations of interfering (S^−^ and F^−^) ions.

## 3. Pyrazole derivatives in molecular sensing

As the chemistry of some molecular species plays a significant role in the metabolic pathways of living organisms, in industrial applications, and in environmental issues, detecting the presence and concentration of certain important compounds in solution is necessary for a variety of medicinal and industrial contexts as well as national security concerns of many countries; consequently, fast, easy and precise quantification of molecules is needed. Therefore, molecular detection and quantification using the chemosensor approach is a growing research field, and pyrazole-based probes have been broadly applied in this area.

### 3.1. Explosives (picric acid)

**Fig. 16:**
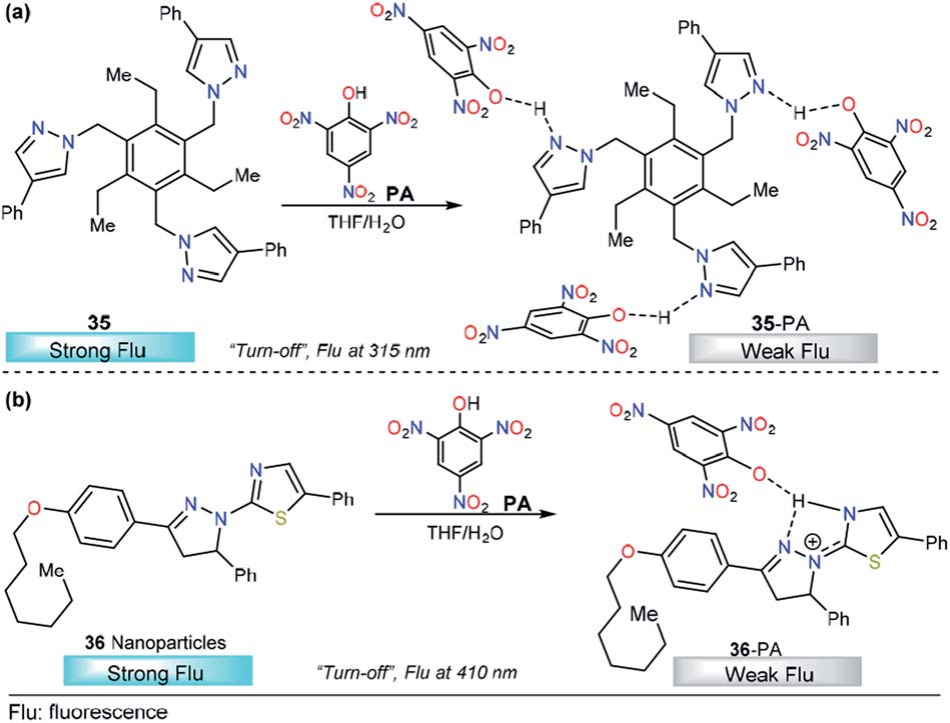
Probes for picric acid based on pyrazoles (a) 35 and (b) 36.

Rapid detection of explosives such as trinitrotoluene (TNT) and picric acid (PA) is important owing to their broad applications including tactical and humanitarian demining, forensic and criminal investigations, *etc.*^153^ As a result, a number of sensing approaches have been developed for their analysis using carbon dots,^154^ fluorescent membranes,^155^ fluorescent oligomers,^156^ and metal–organic frameworks (MOFs),^157^ among others. In particular, PA has relevance in dye industries, pharmaceuticals, and chemical laboratories. Thus, the quantification of this compound has received special attention and many research groups around the world have reported creative solutions. In this context, Raptis and his research team used a tripodal pyrazole derivative 35 with aggregation induced emission enhancement properties for the detection of picric acid (Fig. 16a).^158^ In pure THF, compound 35 is weakly fluorescent, with *λ*_max_ in the 310–330 nm range. After water addition (THF/water 20 : 80), high-intensity fluorescent aggregates were formed. Upon the addition of picric acid, an increase in the absorption band at 360 nm was observed, attributable to charge transfer complex formation and, at the same time, a fluorescence quenching was noticed because of a PET process from electron-rich trispyrazoles in 35 to electron-poor picric acid. This assignment was studied by computational methods at the DFT level and by ^1^H NMR experiments. Crystallographic studies confirmed the formation of 35–PA complexes in which the pyrazole unit plays an essential role in strong N–H binding. Chemodosimeter 35 has a LOD of 450 ppb, comparable to those reported earlier by Roy *et al.*^159^ Further studies revealed the applicability of 35 in the detection of picric acid vapors and in contact mode by applying small spots finding the detection capability of 35 to be at the nanomolar level.

Another interesting example of pyrazole derivatives for PA sensing was published by M. Ahmed and coworkers. This time, fluorescent thiazole-substituted pyrazoline nanoparticles (36) were prepared and tested with a group of nitrocompounds (Fig. 16b).^160^ The UV-vis spectrum of nanoparticle 36 recorded in DMF/H_2_O (1 : 99, v/v) revealed a slight peak broadening and blueshift as compared to that of compound 36–PA in DMF, which showed a maximum absorption at 351 nm. Importantly, the fluorescence emission spectrum (*λ*_exc_ = 361 nm) of nanoparticle 36 in DMF/H_2_O (1 : 99, v/v) showed an enhancement in fluorescence intensity. Remarkably, a substantial reduction in the fluorescence intensity was achieved with PA in comparison with other nitrocompounds tested, and when the amount of PA increased, a regular, linear decrease in the emission of 36 was observed (from 0 to 6 μM). Furthermore, a very low LOD (2.0 ppb) was observed. The increased selectivity of 36 toward PA was attributed to the high acidity of the PA compared with other nitrocompounds and phenol. When complex 36–PA forms, an electron transfer takes place, deactivating the excited state, and as consequently, a lower emission intensity is displayed. As shown in Fig. 16b, the nitrogen atom of the pyrazoline is very important for the selectivity of 36 toward PA.

### 3.2. Biomolecules

**Fig. 17:**
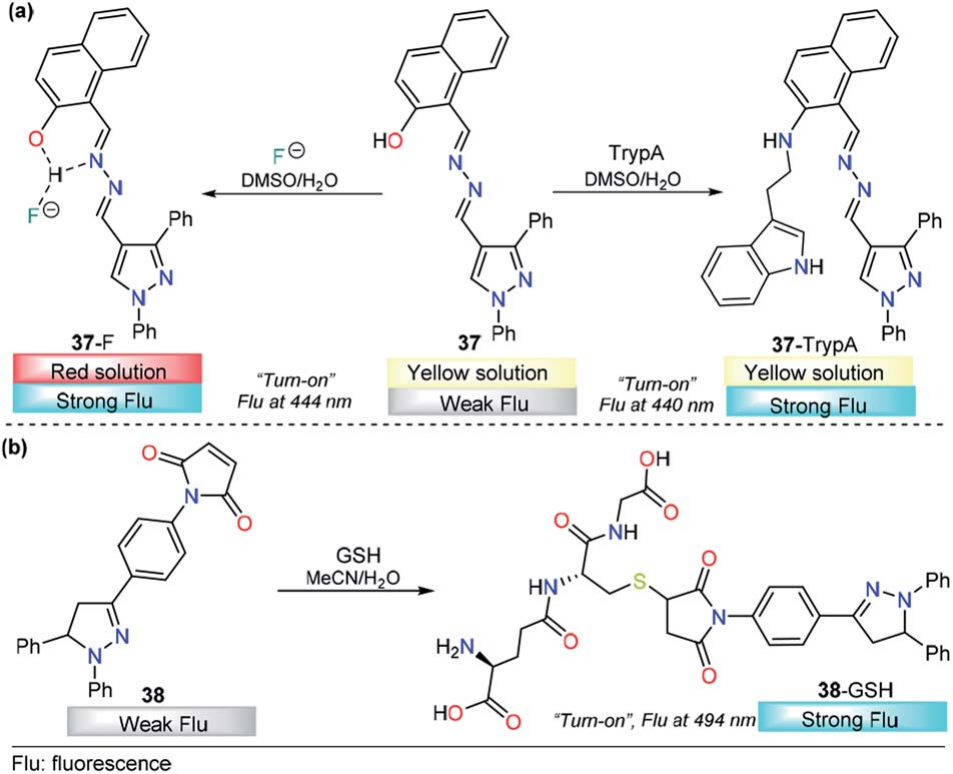
Probes for tryptamine based on pyrazoles (a) 37 and (b) 38.

Tryptamine (TryptA) plays a substantial role as a neurotransmitter and neuromodulator and is found in trace amounts in the brains of mammals.^161,162^ Several analytical methods have been employed in TryptA quantification, but those methods have been shown to be invasive and difficult to adopt in biological samples. Pursuing an approach with the advantages of the pyrazole systems, Siva and his research team proposed the new probe 37 to detect TryptA *via* fluorescence measurements (Fig. 17a).^163^ This probe was equipped with a pyrazolic ring attached to a naphthyl group by a hydrazone bridge. The photophysical properties of 37 were investigated in DMSO/water (9 : 1, v/v) by using a variety of amines such as methylamine, dimethylamine, trimethylamine, phenylethylamine, azane, spermidine, spermine, tyramine, cadaverine, putrescine, serotonin, dopamine, histamine, glycine, l-tryptophan, and d-glucosamine. The absorption spectrum of 37 presented three bands centered at 250 nm, 333 nm and 383 nm. Under identical conditions, only upon the addition of TryptA was a noticeable absorbance change observed. A new band appeared at 282 nm along with a slight decrease in the intensity of the band at 383 nm. Similar results were obtained when fluorescence experiments were performed. This time, TryptA showed a remarkable “*turn-on*” response with an increase in the fluorescence intensity of the enol band at 440 nm accompanied by the disappearance of the keto tautomer band at 503 nm. By using fluorescence measurements, the authors found an extraordinarily low LOD (4.0 pM).

**Fig. 18:**
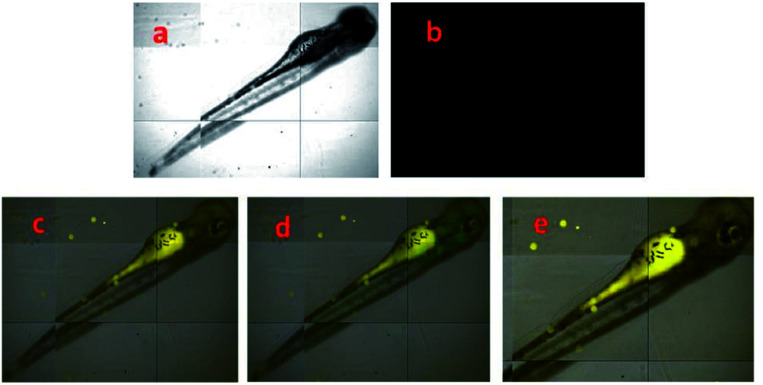
TryptA fluorescence imaging analysis in 4 days old zebrafish embryos fed with different concentrations of TryptA (a) bright field images of pre-treated TryptA (50 μM), (b) fluorescence merged images of pre-treated TryptA (c) 10 μM of TryptA (d) 25 μM of TryptA (e) 50 μM of TryptA for 2 h followed by incubation with 37 (50 μM) for 1 h. Reprinted with permission from ref. 163. Centre National de la Recherche Scientifique (CNRS) and RSC.

Additionally, due to the acidity of the proton in the hydroxyl group in the pyrazole 37, this probe could be used as a selective fluoride chemodosimeter. Upon the addition of F^−^ ions, the color of 37 changed from yellow to orange, the absorbance band at 384 nm diminished with increasing addition of F^−^ ions; next, a new band appeared at 480 nm, with a clear isosbestic point at 422 nm. Fluorescence titration (DMSO/water 9 : 1, v/v) was performed in the presence of many anions, where the addition of F^−^ ions to the probe caused an increase in the intensity of the emission bands at 444 nm and 501 nm. The biological application potential was tested, and the results showed an excellent performance of 37 in live cell imaging analysis of TryptA and F^−^ ion in human HeLa cells and zebrafish embryos (Fig. 18).^163^ A plausible mechanism for TryptA and F^−^ ions detection was proposed by the authors. In both cases, probe 37 showed excellent fluorescence enhancement by inhibition of the excited-state intramolecular proton transfer process. A nucleophilic substitution followed by elimination of the –OH group leads to the 37–TryptA adduct, which was confirmed by ESI-MS and ^1^H NMR studies. On the other hand, the coordination mode of 37–F^−^ was evidenced from the Job plot, ^1^H NMR experiments and ESI-MS analysis. In this case, the pyrazolic ring was used as a structural component that had no influence either on the substrate binding of 37 or on its photophysical properties.

Glutathione (GSH) plays a major role in controlling the disulfide–thiol equilibrium in biological systems. Furthermore, GSH is used as a pharmaceutical compound and can be used in food additives and the cosmetic industries.^164^ Hence, the detection of GSH in the biological sample is of great interest in the field of biochemistry and medicine. Since conventional methods are not suitable for practical use, because they are invasive and destructive methods, the *off–on* fluorescent strategy could be a versatile tool for develop alternative methods for GSH sensing, and with this purpose Subramaniyan and coworkers designed and synthetized the fluorescent probe 38 (1,3,5-triphenylpyrazoline) for GSH sensing (Fig. 17b).^165^ Emission properties responses of 38 toward a pool of nucleophilic species, including cysteine, thioglycolic acid, mercaptoethanol and mercaptopropionic acid, were carry out in MeCN/water; 1 : 9. It was noted that 38 showed strong fluorescence with the emission band at 494 nm, whereas other nucleophilic species, anions or molecules, showed negligible fluorescence. The probe 38 shows to be highly selective for GSH in the presence of other related compounds and has an excellent performance in a wide range of pH 2–12. A linear relationship was found between 38 fluorescent intensity and GSH concentration and a LOD of 0.06 μM. The suitability of 38 was verified in real biological samples such as artificial cerebrospinal fluid, artificial urine and human serum. Moreover, a non-invasive method for bio-imaging GSH in the live cells under oxidative stress based on “*turn-on*” fluorescence of probe 38 was demonstrated. The intrinsic fluorescent properties in substituted pyrazolines is a key factor in the performance of 38. Under nucleophilic attack, the photoinduced electron transfer from pyrazoline to maleimide group is blocked and an activation of the fluorescence is observed.

## 4. Biological applications

Biological imaging of specific molecules or ions can provide direct information on molecular and ion functions in living systems. The most important breakthrough for this purpose is to create selective and sensitive sensing tools. However, for practical applications some parameters should be considered such as the effective concentration range and the kinetic parameters (association and dissociation rates constants), which are important.^166^ In addition to these chemical parameters, biological variables, for example, cell-membrane permeability, intracellular localization, and the toxicity of the excitation light and the sensor molecules themselves to the cells, need to be considered.^167^ Throughout this review, we have highlighted the preliminary applications of pyrazole derivative probes, and some of them have been shown to meet the aforementioned requirements. Accordingly, we have summarized the properties of pyrazole-based probes that have been applied in this area (Table 1).

**Table 1:** Chart of important features of the reported pyrazole-based probes discussed above

Analyte (interference)	Probe (ref.)	*λ*_exc/_ *λ*_em_ (nm)	LOD	Solvent (*K*, M^−1^)	Cell line	Cell viability (cytotoxicity)
Cu^2+^ (Ni^2+^)	4	376/491	43 nM	Ethanol (5.24 × 10^3^)	MG-63	
Zn^2+^	7	380/467	0.295 nM	Ethanol/water (1.47 × 10^4^)	BHK-21	100 μM → 85%
	8	382/471	0.160 nM	Ethanol/water (2.38 × 10^3^)	BHK-21	
	9	323/408	2.90 nM	Ethanol/water (1.88 × 10^4^)	N2A	IC_50_ 37.10 μM
Hg^2+^	12	374/438	9.20 nM	DMSO/water (7.1 × 10^5^)	HepG2	1.0 μM → 99%
Al^3+^	15	380/470	4.29 nM	Water (1.76 × 10^5^)	HepG2	4.0 μM → 80%
	16	400/468	62.0 nM	Water (—)	HeLa (lysosomes)	
	17	300/450	1.20 nM	DMSO/water	HepG2	
	18	418/518	—	CH_3_CN/water (2.13 × 10^3^)	Mouse embryonic fibroblast 3T3-L1	
	19	330/495	81 nM	Ethanol/water (1.89 × 10^3^)	HeLa	
Cr^3+^	21	446/506	37 pM	DMSO (3.38 × 107 dm^3^ mol^−1^)	A-549	
Tryptamine (F^−^)	37	334/440	0.04 nM	DMSO/water (2.17 × 10^−2^)	Zebra fish	50 μM → 94%
Glutathione	38	360/494	60 nM	CH_3_CN/water (—)	aCSF^a^, aU^b^, human serum, *E. coli*^c^	

a aCSF: artificial cerebrospinal fluid.

b aU: artificial urine.

c *E. coli*: *Escherichia coli*.

Table 1 shows that pyrazole-based probes represent an interesting alternative for biological applications using fluorescence confocal microscopy. The biological and chemical parameters achieved are as follows: (i) most of them are studied in highly polar solvents and are compatible with water. (ii) The LODs are at the nanomolar level. (iii) Fluorescent bioimages can be obtained from a variety of cells, showing their cell-membrane permeability and intracellular localization. However, additional studies need to be performed in order to visualize the real application of these probes, for example, estimating the cytotoxicity of the cells with the wavelength and solvent used and making a comparison of the association constant of the probe-analyte complex with respect to biological ligands (*e.g.*, enzymes)-analyte. The wavelength of excitation needs to be improved in order to avoid the possible cytotoxicity of cell media under UV-vis irradiation. Finally, the LOD achieved need to be compared with the concentrations found at the cellular level.

## 5. Conclusions and perspectives

It is amazing what a small molecule scaffold such as substituted pyrazole and its derivatives have achieved. The abundance of research aimed at finding ways to utilize these compounds to replace some heterocyclic rings that are not as efficient is justified by many recent publications describing their unique properties, together with the versatility of their synthesis and chemical modification. However, it is important to note that several works describing pyrazole-based chemosensors do not have a rational design. Therefore, this review can be seen as a helpful contribution to this important research field.

The synthesis and application of fluorescent small-molecule ion/molecules sensors is a viable approach to probe the cell biology of these species and has yet to reach its full potential. In these areas, pyrazole derivatives have been involved in the detection and quantification of ions of different natures, including size, charge, magnetic properties, toxicity and availability, such as Cu^2+^, Zn^2+^, Hg^2+^, Al^3+^, Fe^3+^, Cr^3+^, and F^−^, and essential metabolites such as glutathione and tryptamine. Likewise, other species with industrial, environmental and national security relevance such as Ag^+^, CN^−^ and picric acid have also been subject the of research by using this kind of probe. The mechanisms by metal sensing have been explained, and a variety of processes take place during analyte quantification, including PET, ICT, ESIPT and CHEF processes, among others.

Pyrazole-based probes represent an interesting tool in the field of small-molecule chemosensors/chemodosimeters due to the following key characteristics: the particular structural orientation of pyrazoles, the direct involvement of the moiety in cation coordination (through the pyridine-type nitrogen atom), the amphoteric behavior when participating in acid–base reactions, with anions or highly acidic organic compounds, or simply the ability to take advantage of its intrinsic fluorescent properties. Furthermore, the pyrazole-based probes that have been utilized for the detection of analytes inside cellular media *via* confocal fluorescence microscopic means have been mentioned in the description of each sensing strategy.

Although the more representative example for Cu^+^ sensing is based on a pyrazoline derivative,^168^ the recent pyrazole probes for the detection of copper are only selective for the divalent species (Cu^2+^). Thus, to meet the requirements for biological applications,^169^ research needs to be done in this direction. Notably, since Cu^+^ has different magnetic properties from those of Cu^2+^, a more desired “*turn-on*” behavior can be expected. Likewise, owing to structural properties such as the disposition of heteroatoms and photophysical tuning, the combination of pyrazole with pyridine is the most common in pyrazole-based probes for cation sensing. However, the substitution position needs to be carefully choose to obtain the best results and take advantage of the chelating properties of the molecular probe.

On the other hand, the anion sensing performance of pyrazole derivatives finds an optimal response when a fused pyrazole is used. The intrinsic fluorescent properties and π-extended conjugation of these derivatives have become attractive tools in probe design for anions. However, none of the examples discussed here show reversible properties, which is a disadvantage in this area. Consequently, for the design of new fluorophores, it is necessary to take into account the need for reuse of these materials to avoid the generation of waste.

## Conflicts of interest

The authors declare no competing financial interest.

## Supplementary Material
